# Quantitative systems pharmacology modeling of HER2-positive metastatic breast cancer for translational efficacy evaluation and combination assessment across therapeutic modalities

**DOI:** 10.1038/s41401-024-01232-9

**Published:** 2024-02-15

**Authors:** Ya-ting Zhou, Jia-hui Chu, Shu-han Zhao, Ge-li Li, Zi-yi Fu, Su-jie Zhang, Xue-hu Gao, Wen Ma, Kai Shen, Yuan Gao, Wei Li, Yong-mei Yin, Chen Zhao

**Affiliations:** 1 Department of Oncology, The First Affiliated Hospital of Nanjing Medical University, Nanjing, 210029 China; 2 Department of General Surgery, The First Affiliated Hospital of Nanjing Medical University, Nanjing, 210029 China; 3 Gusu School, Nanjing Medical University, Suzhou, 215000 China; 4 School of Pharmacy, Nanjing Medical University, Nanjing, 211166 China; 5 Department of Breast Disease Research Center, The First Affiliated Hospital of Nanjing Medical University, Nanjing, 210029 China; 6 Jiangsu Hengrui Medicine Co. Ltd, Shanghai, 200245 China; 7 QSPMed Technologies, Nanjing, 210000 China

**Keywords:** quantitative systems pharmacology, HER2^+^ metastatic breast cancer, therapeutic combinations, drug resistance, model-informed drug development

## Abstract

HER2-positive (HER2^+^) metastatic breast cancer (mBC) is highly aggressive and a major threat to human health. Despite the significant improvement in patients’ prognosis given the drug development efforts during the past several decades, many clinical questions still remain to be addressed such as efficacy when combining different therapeutic modalities, best treatment sequences, interindividual variability as well as resistance and potential coping strategies. To better answer these questions, we developed a mechanistic quantitative systems pharmacology model of the pathophysiology of HER2^+^ mBC that was extensively calibrated and validated against multiscale data to quantitatively predict and characterize the signal transduction and preclinical tumor growth kinetics under different therapeutic interventions. Focusing on the second-line treatment for HER2^+^ mBC, e.g., antibody-drug conjugates (ADC), small molecule inhibitors/TKI and chemotherapy, the model accurately predicted the efficacy of various drug combinations and dosing regimens at the in vitro and in vivo levels. Sensitivity analyses and subsequent heterogeneous phenotype simulations revealed important insights into the design of new drug combinations to effectively overcome various resistance scenarios in HER2^+^ mBC treatments. In addition, the model predicted a better efficacy of the new TKI plus ADC combination which can potentially reduce drug dosage and toxicity, while it also shed light on the optimal treatment ordering of ADC versus TKI plus capecitabine regimens, and these findings were validated by new in vivo experiments. Our model is the first that mechanistically integrates multiple key drug modalities in HER2^+^ mBC research and it can serve as a high-throughput computational platform to guide future model-informed drug development and clinical translation.

## Introduction

Approximately 15% to 20% of breast cancer patients have significant overexpression of human epidermal growth factor receptor 2 (HER2), and these patients are reported to be associated with aggressive tumor growth and poor prognosis [1, 2]. Over the past 25 years, advances in HER2-targeted therapies have dramatically improved the outcomes of patients with HER2^+^ metastatic breast cancer [1]. Among these, monoclonal antibodies (mAb) such as trastuzumab and pertuzumab, small molecule tyrosine kinase inhibitors (TKI) such as lapatinib and pyrotinib, and antibody-drug conjugates (ADC) such as trastuzumab emtansine (T-DM1) and trastuzumab deruxtecan (T-DXd), are the three main therapeutic modalities for HER2^+^ mBC. The current first-line treatment according to NCCN is dual HER2 blockade with trastuzumab and pertuzumab combined with chemotherapy [3], followed by HER2 ADCs (T-DXd or T-DM1) as second-line based on the results of the DB-03 [4] and EMILIA studies [5]. In China, pyrotinib (an irreversible pan-ErbB TKI) in combination with capecitabine is currently recommended as a preferred option for second-line treatment according to the PHOEBE study [6]. Despite these achievements, there are still many issues to be addressed in treating HER2^+^ mBC, such as the potential of new drug combinations, feasibility of new dosing regimens, various mechanisms of resistance, as well as clinical value of new emerging drug targets. Therefore, drug research and development are still in full swing in this field as evidenced by the large number of clinical trials actively going on [7].

HER2 is a receptor tyrosine kinase that belongs to the epidermal growth factor receptor family (ErbB family). This family comprises four members, including EGFR (HER1/ErbB1), HER2 (ErbB2/neu), HER3 (ErbB3) and HER4 (ErbB4). They each consist of an extracellular ligand-binding domain, a transmembrane domain and an intracellular tyrosine kinase catalytic domain [8]. The ligands for ErbB receptors include epidermal growth factor (EGF), amphiregulin (AR) and betacellulin (BTC) which mainly bind to EGFR, and neuregulin1 (NRG1, also called heregulin), which mainly binds to HER3 and HER4 [9]. Ligand binding to the extracellular domain induces a conformational change that allows homo- or heterodimerization and subsequent transphosphorylation [10]. Notably, HER2 has no known ligand [11], whereas HER3 is defective in tyrosine kinase activity [12] so that HER3 homodimer is nonfunctional. Phosphorylated receptors then activate downstream signaling pathways such as Ras/MAPK, PI3K/AKT, PLC-γ and STAT [10], which regulate cellular processes under normal conditions but are associated with uncontrolled cellular activities due to aberrant ErbB signaling when receptors are overexpressed, ultimately leading to tumorigenesis and tumor growth [13]. HER2^+^ BC is one such example. Overexpression of HER2 biases dimer formation toward HER2-containing homo- and heterodimers (particularly HER2/HER2 and HER2/HER3 complexes) [14], resulting in constitutive activation of downstream pathways in a ligand-independent manner [15, 16]. In addition, HER2 heterodimers can also be activated in ligand-dependent manners during EGF and NRG1 stimulation [13]. In the two major downstream pathways PI3K/AKT and Ras/MAPK, multiple feedback regulatory mechanisms are also implicated in dynamically driving the pro-tumorigenic ErbB network. For example, in the HER2-HER3 axis, inhibition of HER2 results in decreased AKT activation which can upregulate HER3 production through enhanced FOXO3a transcriptional activity, and this may compensate pro-survival signaling and is a reason for the failure of HER2-targeted therapies [17, 18]. Due to the complexity of ErbB network and multifaceted features of HER2^+^ BC, exploring combination therapy is an important direction of current research. Therapeutic potential of combinations between HER2-targered therapies (e.g., ADC and TKI) [19, 20] or with agents of other mechanisms of action such as inhibitors of PI3K [21], ERK [22], and immunotherapy [23] is being actively investigated with many combinations already in clinical trials.

Since the ErbB signaling system is a highly complex dynamic network and plays an important role in breast cancer, mechanistic modeling will help us better understand the system and guide more effective therapeutic designs. Birtwistle et al. developed a model describing the short-term activation of ERK and AKT in MCF-7 breast cancer cells and used the model to explain differential effects of ERK inhibition [24]. Another work by Schoeberl et al. contains a pool of ErbB ligands and through model sensitivity analysis they identified HER3 as the most critical node affecting AKT signaling [25]. Vaidya et al. constructed a PK/PD model focusing on the PI3K/AKT pathway to evaluate the cytotoxicity of a specific triple combination therapy against JIMT-1, a HER2-therapy-resistant cell line [26]. The above works uniquely demonstrated the advantage of using computational models in deciphering disease-driving mechanism and drug targets; however, regarding the treatments for HER2^+^ mBC, the latest therapeutics (e.g., HER2-ADC, pyrotinib) have not been considered nor their mechanisms included, which means that a gap still exists for model-informed translation. Thus, we here developed a new mechanistic quantitative systems pharmacology model that integrates ErbB signaling networks in HER2^+^ BC cells, pharmacokinetics of different state-of-the-art drug modalities, as well as mechanisms of drug-induced cellular perturbations and tumor growth to facilitate drug development and translation. This model accurately reproduces a large set of in vitro cell signaling and dose-viability data as well as in vivo antitumor efficacy data when different drug modalities are administered in mouse xenografts. The model predicted that compared to classic TKI plus capecitabine, a novel combination of TKI plus ADC was more effective in inducing tumor regression even at significantly lower doses, and it also suggested that sequential administration of HER2-ADC followed by TKI plus capecitabine would prolong response duration compared to direct TKI plus capecitabine alone, and both findings were validated by in vivo data. Through model analysis we further identified NRG1-driven signaling compensation as a mechanism of TKI resistance, and the addition of HER3 antibodies could effectively reverse it. This model provides a unique platform to simulate the potential therapeutic effects of different drug combinations and regimens as well as identify mechanisms of drug resistance and possible solutions, which lay the foundation for subsequent virtual patient development and clinical trial simulations in HER2^+^ mBC.

## Materials and methods

### Model formulation, parameter estimation and in silico pathophysiological simulation

The model was constructed based on ordinary differential equations (ODEs) using mass-action laws and Hill-type functions. A total of up to sixty species and eighty reactions were included in the system (including all PK components). Cellular biological behaviors of the ErbB system were described mainly by mass-action laws, from receptor-ligand binding to receptor dimerization, phosphorylation and degradation, then to activation of downstream signaling pathways, while signal transduction, feedback loop, and tumor growth were described by Hill functions. Five clinically approved therapeutics including T-DM1, T-DXd, lapatinib, pyrotinib and capecitabine were incorporated into the model and their PK profiles were characterized using standard ODE-based compartment models [27–29]. To characterize the concentrations of drugs entering the tumor microenvironment, we made a simplified assumption that drug exposures in the tumor are proportional to that in the blood, as described by tumor partition coefficients for TKIs and capecitabine. For the two ADCs, we assumed that they could enter tumor microenvironment at a constant rate from central compartment. Mechanisms of action of two TKIs were modeled as inhibiting phosphorylation of their target receptors and inducing receptor degradation, whereas T-DM1, T-DXd and capecitabine were modeled as inducing tumor death.

For model calibration at the cell level, we primarily used experimental data from the SKBR3 breast cancer cell line. The number of EGFR, HER2, HER3 and HER4 receptors per cell in SKBR3 is 150,000, 1,500,000, 40,000 and 2000 respectively as derived from quantitative literature measurements [30–32]. Since HER2 can form homodimers or heterodimers with HER3 spontaneously in the absence of any ligands, the model species will reach a steady state and we use the steady-state concentrations as the new initial condition. To evidence the generality of the model, calibration procedures were also performed in other three cell lines (BT-474, NCI-N87 and ZR-75-1 with HER2 IHC levels of 3+ , 2+ and 1+ , respectively [33–35]) by varying the initial amount of HER2, the steady-state concentrations of the model species, and parameters related to tumor cell growth (n13, km13) and death (w15, km10, w14, n14 and km14), with the assumption that there are 1.5 million HER2 receptors per cell in IHC 3+ , 0.5 million in 2+ and 0.1 million in 1+ cell lines as measured by previous in vitro studies [30, 36]. Initial conditions of model species and parameter values in different cell lines along with their descriptions are summarized in Supplementary Table S1.

For in vivo translation of the model, tumor growth kinetics data in SKBR3-derived mouse xenografts were primarily used. Baseline in vivo tumor proliferation and death rates (umax and dmax) were re-estimated using experimental data from lapatinib- and pyrotinib-treated SKBR3 xenografts. For drug-induced in vivo cell death, considering data availability, we used published data from KPL4 (a HER2+ BC cell line with IHC 3+ as well) xenografts whose tumor growth curve in the control arm was consistent with that in lapatinib- and pyrotinib-treated SKBR3 models (e.g., with the same umax and dmax) to further estimate the parameter of capecitabine-induced max stimulation of tumor cell death (w15). T-DM1-treated tumor growth kinetics were also obtained from KPL4 xenografts yet showing a slightly different growth trend in the control group. Therefore, for this we recalibrated the basal tumor proliferation and death rates (umax and dmax), estimated the T-DM1-mediated max stimulation of tumor death parameter (w14) on this basis and took this value for subsequent model simulations. Similarly, tumor growth kinetics upon T-DXd treatment in a HER2-overexpressing PDX model were used to estimate T-DXd-induced max stimulation of tumor death parameter (w14). Also, to evidence the generality of the model, we performed the same in vitro to in vivo translation strategy in BT-474 (another HER2 3+ BC cell line) xenografts. By correspondingly adjusting the proliferation and death rates (umax and dmax) and parameters related to drug-induced tumor cell death (w14, n14 and km14), the model successfully and quantitatively captured all the time-dependent tumor growth kinetics under lapatinib, pyrotinib and T-DM1 treatments measured in BT-474 CDX mice. It should be noted that the aforementioned parameters were simultaneously fitted in the control and treatment groups. When comparing our model simulations to in-house data obtained from mouse experiments, we only adjusted umax to match the tumor growth in the control group, and then tumor growth kinetics in all the drug treatment groups were simulated without any further parameter changes for model validation purposes.

The Bliss independence principle was applied to determine the combination effect of two drugs using the equation *Yab* = *Ya* + *Yb* − Ya * Yb, where *Yab* is the Bliss predicted combination effect of drugs A and B at given doses and *Ya*/*Yb* denote the effects of drug A/B alone at dose *a/b* respectively [37]. If the model simulated effect at a certain dose pair of drug A and drug B is greater than the Bliss predicted effect *Yab*, the drug combination is regarded as having synergistic effects at that specific dose combination.

ImageJ software (NIH, Bethesda, MD, USA) was used for Western blot densitometry band quantification. For signal transduction and drug intervention fittings, all values are relative protein expression levels, either normalized to their respective maximum values or normalized to their respective control condition. For cell viability fittings, we added different concentrations of drugs to the model for a certain period of time to simulate changes in total cell amount, and then we normalized the resulting cell amount values to the untreated conditions undergoing the same period of time to obtain the simulated cell viability curves. All model reactions were implemented in MATLAB Simbiology Toolbox (MathWorks, Natick, MA, USA), and simulations and analyses were performed using the ode15s solver. Parameter values were determined from the experimental data and published models (e.g., association/dissociation rates, receptor half-lives) as well as from data fitting. For parameter estimation and optimization, the *patternsearch* function in global optimization toolbox was used. A summary of all model species, parameters, reactions, and their descriptions, along with a spreadsheet summarizing the data used for calibration versus validation is provided in Supplementary Table S1.

### Global sensitivity analyses

The Partial rank correlation coefficient (PRCC) algorithm was performed according to the methodology introduced by Marino et al. [38]. For the algorithm settings, we used 5000 iterations with Latin Hypercube Sampling for each input condition and parameter value ranges were set to one-half to two-fold of their baseline in the PRCC calculations. Tumor volume at day 20 (from day 0) was the output of interest for all PRCC calculations. A total of six different input conditions were analyzed, including no external stimuli, NRG1 overexpression, lapatinib plus capecitabine, pyrotinib plus capecitabine, single agent T-DM1 and single agent T-DXd. Parameters significantly correlated with the model output under each condition (e.g., PRCC value greater than 0.05 or less than −0.05 with statistical significance) were presented separately. To verify the PRCC results, Sobol sensitivity analysis [39] was further conducted under the conditions of no external stimuli, NRG1 overexpression, lapatinib plus capecitabine, and single T-DM1, with the same set of parameters and model output as for PRCC. Total-order Sobol indices of each parameter at the end of simulation were computed and analyzed.

### Uncertainty analysis

We selected a total of 20 top-ranked parameters from six runs of sensitivity analyses mentioned above to perform parameter uncertainty analysis. For that, the optimization datasets were resampled 50 times based on numerical ranges defined by the corresponding datapoint mean values and standard deviations or standard errors that we collected (for datapoints with only mean values available, we assumed that their standard deviations equaled 10% of the mean values). The optimization algorithm (as described above) was then performed using the 50 resampled datasets to obtain 50 sets of new parameter estimates for the 20 parameters, and the parameter values were allowed to vary from one-tenth to ten-fold of their baseline during bootstrapping. The final readout of the uncertainty analysis is the relative value distribution of the 20 selected parameters.

### Cell line and chemical reagents

Human breast cancer cell line SKBR3 was obtained from American Type Culture Collection (ATCC, Manassas, VA, USA) and cultured in high-glucose Dulbecco’s modified Eagle’s medium (KGL1206-500, KeyGEN, Nanjing, China) supplemented with 0.1 mg/mL streptomycin, 100 IU/mL penicillin, and 10% fetal bovine serum (C04001-500, Vivacell, Shanghai, China) at 37 °C and 5% CO_2_ humidified environment. All the chemical reagents and antibodies used in this study are listed in Table 1.

**Table 1:** Chemicals and antibodies used in this study.

List	Chemical/antibodies	Dilution	Vendors (District)	Catalog No.
Chemical	EGF	100 ng/mL	MCE (Shanghai, China)	HY-P7109
(in vitro)	Pyrotinib	5, 10, 20, 40, 80 nmol/L	MCE (Shanghai, China)	HY-104065
	NRG1	50 ng/mL	Cell Signaling (Danvers, MA, USA)	26941
Chemical	Lapatinib	as directed	MCE (Shanghai, China)	HY-50898
(in vivo)	Capecitabine	as directed	MCE (Shanghai, China)	HY-B0016
	Pyrotinib	as directed	MCE (Shanghai, China)	HY-104065
	T-DM1	as directed	MCE (Shanghai, China)	HY-P9921
Antibodies	HER2	1:1000	Abcam (Cambridge, UK)	ab237715
	HER3	1:1000	Proteintech (Wuhan, China)	10369-1-AP
	pHER3	1:10,000	Abcam (Cambridge, UK)	ab133443
	pHER4	1:10,000	Abcam (Cambridge, UK)	ab109273
	pEGFR	1:400	Abcam (Cambridge, UK)	ab223499
	pERK	1:10,000	Abcam (Cambridge, UK)	ab278538
	Goat anti-rabbit IgG H&L (HRP)	1:50,000	Abcam (Cambridge, UK)	ab205718
	GAPDH	1:40,000	Proteintech (Wuhan, China)	10494-1-AP

### Western blot

SKBR3 cells were seeded in 12-well plates at a density of 3 × 10^5^ cells per well, and allowed for attachment for 24 h. For dose response, SKBR3 cells were treated with EGF protein (100 ng/mL) and pyrotinib at different doses (0, 5, 10, 20, 40, 80 nmol/L) simultaneously for 60 min. For NRG1 plus pyrotinib treatments, cells were exposed to NRG1 protein (50 ng/mL) and pyrotinib (0, 5, 10, 20, 40, 80 nmol/L) at the same time for 60 min. For time course analyses, cells were treated with pyrotinib (20 nmol/L) for 0, 1, 3, 6, 12, 24 h. Then, cells were washed with pre-cold PBS buffer (KGL2206-500, KeyGEN, Nanjing, China) for three times, and then lysed and harvested in RIPA lysis buffer (KGB5205-100, KeyGEN, Nanjing, China) containing 50× protease and phosphatase inhibitor cocktail (P1046, Beyotime, Shanghai, China). The supernatants were collected after centrifugation at 12,000 × *g* at 4 °C for 10 min. Protein concentration was determined using a BCA protein quantification kit (E112-01, Vazyme, Nanjing, China). Protein samples were mixed with 5× SDS loading buffer (G2013, Servicebio, Wuhan, China) and heated at 100 °C for 10 min, and then separated by 8% or 10% SDS-PAGE gel (E302, E303, Vazyme, Nanjing, China) and transferred onto a pre-wetted polyvinylidene fluoride membrane (Immobilon-P PVDF-Membrane, IPVH00010, Merck Millipore, MA, USA) at low current overnight. The membrane was sealed by 4% bovine serum albumin (BSA, BS114, Biosharp, Hefei, China) at room temperature for 1 h and incubated with primary antibodies at 4 °C overnight followed by 1 h incubation with secondary anti-rabbit IgG antibody. Signals were detected with enhanced ECL reagents (E412, Vazyme, Nanjing, China). GAPDH served as a loading control.

### Animal studies

A total of 1 × 10^7^ SKBR3 cells in 100 μL PBS were injected subcutaneously into the right mammary fat pads of the BALB/c nude mice, respectively. When the tumor volume reached nearly 80 mm^3^, mice were randomly assigned into 5 treatment groups (*n* = 5 each): PBS (100 μL, oral gavage, d1–d14 and 100 μL, intravenous injection, d1, d8), lapatinib (100 mg/kg, oral gavage, d1–d14) + capecitabine (200 mg/kg, oral gavage, d1–d14), T-DM1 (10 mg/kg, intravenous injection, d1) + pyrotinib (10 mg/kg, oral gavage, d1–d14), T-DM1 (10 mg/kg, intravenous injection, d1) followed by lapatinib (100 mg/kg, oral gavage, d8–d14) + capecitabine (200 mg/kg, oral gavage, d8–d14), lapatinib (100 mg/kg, oral gavage, d1–d7) + capecitabine (200 mg/kg, oral gavage, d1–d7) followed by T-DM1 (10 mg/kg, intravenous injection, d8). Tumor volumes were measured every three days with a caliper and calculated using the formula [width^2^ × length]/2. At the termination of the experiment (on d15), the mice were sacrificed and the tumors were removed. The weight of each tumor was recorded. The animal experiments were approved by the Scientific and Ethical Committee of the Institute of Nanjing Medical University (approval number IACUC-2311019). BALB/c nude mice (female, 4–5 weeks old) were bought from Zhejiang Vital River Laboratory Animal Technology Co., Ltd (Zhejiang, China) and raised in Animal Core Facility of Nanjing Medical University under specific pathogen-free conditions (Temperature, 20–26 ˚C; humidity, 40%–60%; 12/12-h light/dark cycle; free access to food and water). Animal studies were performed strictly following the Guide for Care and Use of Laboratory Animals of Nanjing Medical University.

## Results

### Overview of the cancer cell model structure

**Fig. 1:**
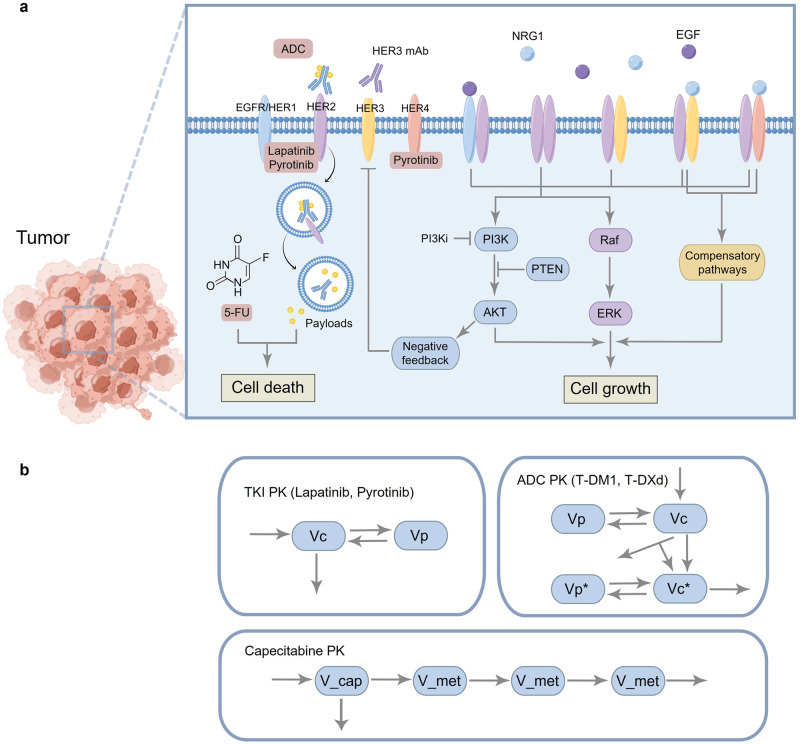
Diagram of the mechanistic model structure. **a** Tumor cell module. At the input level are four ErbB receptors and their respective ligands. Ligand-receptor binding induces dimerization of receptors, activating downstream pathways and regulating cell growth. Transcription factors such as FOXO mediate negative feedback of HER3. Five drugs with unique mechanisms of action are presented and will lead to cell death through different cellular interactions within the system. The model can also simulate drugs for other targets, such as HER3 antibodies, PI3K inhibitors, etc. **b** Pharmacokinetic modules of lapatinib, pyrotinib, T-DM1, T-DXd and capecitabine. Vc and Vp are central and peripheral compartments for TKI (lapatinib, pyrotinib) or ADC (T-DM1, T-DXd). Vc* and Vp* are central and peripheral compartments for payloads of ADC (DM1, DXd). V_cap and V_met are compartments for capecitabine and its metabolites (5’DFCR, 5’DFUR and 5-FU). The overall figure is created by Figdraw and a more detailed model diagram with specific model species and reaction fluxes is shown in Supplementary Fig. S9.

The entire model comprises two parts, including a tumor cell module and multiple pharmacokinetic (PK) modules for drugs (Fig. 1). Based on current knowledge and understanding of HER2+ BC proliferation and survival, we constructed a mathematical model of intracellular signaling in HER2^+^ BC cells to mechanistically explore its relationship with tumor growth. The model incorporates all four ErbB receptors and their respective ligands, with EGF for EGFR and NRG1 for HER3 and HER4. Ligand-bound EGFR, HER3 and HER4 tend to form heterodimers with HER2, as HER2 is the preferred dimerization partner when overexpressed. HER2 can form homodimers in a ligand-independent manner, and we also consider ligand-independent formation of HER2/HER3 heterodimers [16]. Dimerized receptors then undergo transphosphorylation, activating the downstream PI3K/AKT and Raf/MAPK cascades. These signals are ultimately integrated to regulate the growth of tumor cells. Other compensatory pathways can also contribute to tumor growth in certain contexts, for example BTK-C was identified as a survival factor and was involved in NRG1-mediated drug resistance in HER2^+^ BC cells [40]. Besides, feedback of HER3 which can further attenuate the efficacy of pathway inhibitors, was also taken into account [17].

T-DM1, T-DXd, lapatinib, pyrotinib and capecitabine are five representative therapeutic agents (for ADC, TKI and chemotherapy) in HER2^+^ mBC treatment, and their distinct mechanisms of action are physically incorporated into the model. For T-DM1 and T-DXd, when they reach a cell overexpressing HER2, they bind to HER2 and then undergo receptor-mediated endocytosis. After endocytosis and lysosomal processing, the active payloads (DM1 and DXd, respectively) are released into the cytosol where they exert cytotoxic effects and lead to cell death [41]. Besides, the released DM1 can be excreted from the cytosol since it is a substrate for drug efflux pumps such as MDR1 but it cannot re-enter into the cell [42], whereas the released DXd is highly membrane permeable and it can penetrate across the cell freely [43]. For lapatinib and pyrotinib, in addition to the classical mechanisms of inhibiting receptor phosphorylation and thus downstream pathway activation [44], they can also promote receptor ubiquitination and degradation [45]. The difference is that lapatinib mainly targets EGFR and HER2, while pyrotinib can target EGFR, HER2 and HER4. For capecitabine, it must be first metabolized to 5-FU to be tumoricidal. Details of model mechanisms and reactions are available in the Supplementary files.

### Extensive model calibration/validation using cellular signaling and viability data

Model calibration was performed using experimental data measured in SKBR3, a HER2-overexpressing BC cell line with an immunohistochemistry (IHC) level of 3+  [46]. Initial amounts of four ErbB receptors were determined based on the quantitative measurements in SKBR3 cells from literature [30–32]. Since HER2 can form homodimers or heterodimers with HER3 spontaneously in the absence of ligands, the model species will reach a steady state with baseline proliferative signaling on even when no external stimuli are present. On this basis, different stimulus conditions were then used as model inputs to generate a large number of simulations that can describe all respective experimental data simultaneously (a total of approximately 674 datapoints).

**Fig. 2:**
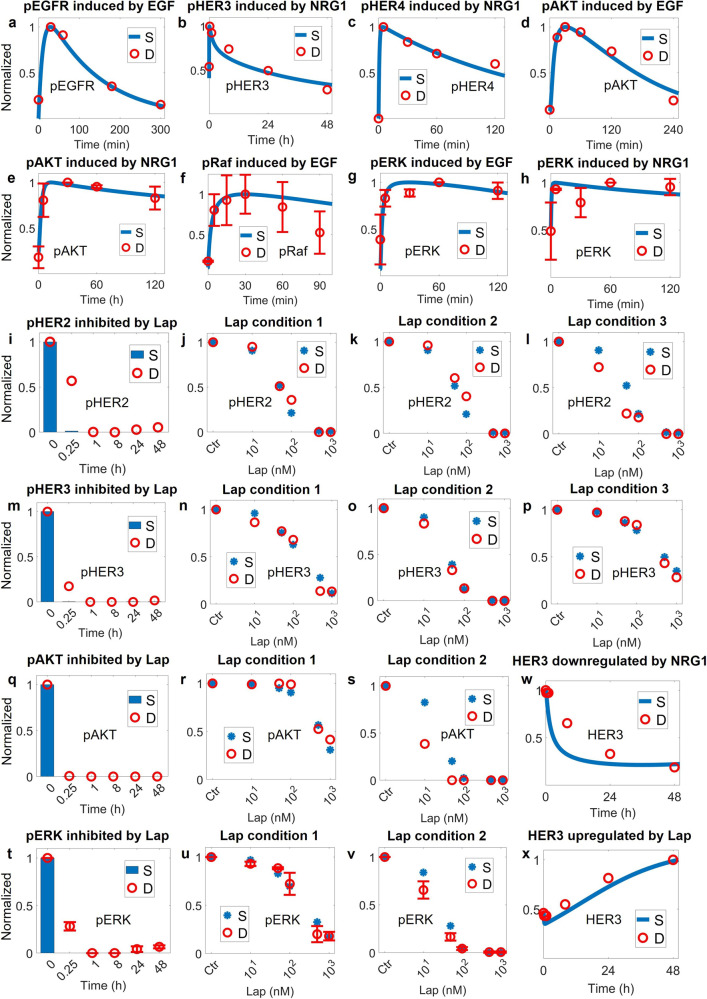
Model calibration of phospho-receptors and downstream PI3K/AKT, Ras/MAPK signal transduction at the cell level. **a** EGF (100 ng/mL) induces activation of EGFR. NRG1 (**b** 50 ng/mL, **c** 200 ng/mL) induces activation of HER3 and HER4. **d** EGF (20 ng/mL) induces activation of downstream AKT. **e** NRG1 (10 ng/mL) induces activation of downstream AKT. EGF (**f** 100 ng/mL, **g** 50 ng/mL) induces activation of downstream Raf and ERK axis. **h** NRG1 (10 ng/mL) induces activation of downstream ERK. Lapatinib induces time-dependent and dose-dependent inhibition of (**i**–**l**) HER2, (**m**–**p**) HER3 and downstream (**q**–**s**) AKT, (**t**–**v**) ERK. **w** NRG1 (50 ng/mL) reduces HER3 expression. **x** Lapatinib induces HER3 expression. **a**–**x** All data are from experiments in the SKBR3 cell line, except in (**c**) (MCF7, HER2 0–1+) and (**f**) (BT20 transfected with ErbB2). Y axes are relative expression levels (normalized to their respective maximum values). Lap condition 1, simultaneous addition of lapatinib and NRG1 (50 ng/mL) for 15 min; Lap condition 2, lapatinib alone for 15 min; Lap condition 3, NRG1 (50 ng/mL) for 15 min followed by lapatinib for 15 min; S, simulation; D, experimental data; Ctr, control/untreated condition.

**Fig. 3:**
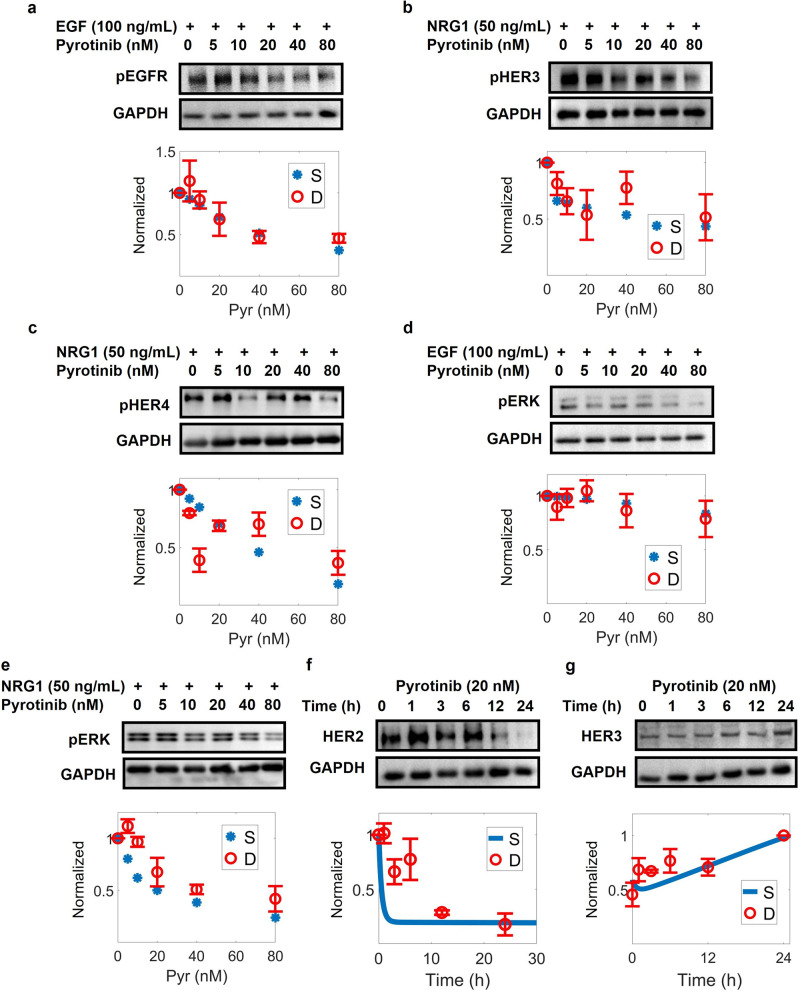
Effect of pyrotinib treatment on ErbB signaling in SKBR3 cells and corresponding model calibration. **a** Dose-dependent inhibition of EGFR after pyrotinib and EGF treatment for 60 min. **b** Dose-dependent inhibition of HER3 after pyrotinib and NRG1 treatment for 60 min. **c** Dose-dependent inhibition of HER4 after pyrotinib and NRG1 treatment for 60 min. **d** Dose-dependent inhibition of ERK after pyrotinib and EGF treatment for 60 min. **e** Dose-dependent inhibition of ERK after pyrotinib and NRG1 treatment for 60 min. **f** HER2 expression in response to pyrotinib treatment. **g** HER3 expression in response to pyrotinib treatment. **a**–**g** Contains immunoblots showing differential regulation of ErbB signaling proteins under various treatment conditions and the immunoblot data (*n* = 3) were quantified and used as calibration data; GAPDH levels were used as controls. Y axes are relative expression levels, with (**a**–**e**) normalized to their respective control condition (e.g., pyrotinib 0 nM) and (**f**, **g**) normalized to their respective maximum values. S, simulation; D, experimental data.

The first level of model calibration was to ensure that our model can accurately reproduce cell signaling data under different ligand stimulation (EGF and NRG1) and drug treatments (lapatinib and pyrotinib) (Figs. 2, 3 and Supplementary Fig. S1). Simulations show that EGF and NRG1 can induce time-dependent activation of EGFR, HER3 and HER4 [47–49] (Fig. 2a–c), respectively, which in turn induces activation of Raf/MAPK and PI3K/AKT pathways [50–52] (Fig. 2d–h and Supplementary Fig. S1a, b, e, f). Lapatinib, as an inhibitor of EGFR and HER2, can induce both time-dependent and dose-dependent inhibition of EGFR, HER2 and HER3 [48] (Fig. 2i–p), thus inhibiting the signaling of both pathways [48] (Fig. 2q–v and Supplementary Fig. S1c, d, g, h). Specifically, our model successfully reproduced the dose-response profiles of phosphorylated receptors and key proteins under three different lapatinib treatment conditions: simultaneous addition of lapatinib and ligands, addition of ligands then followed by lapatinib, and addition of lapatinib followed by ligands. When treated alone, 100 nM of lapatinib was sufficient to inhibit the entire pathway completely [48] (Fig. 2s, v). However, when lapatinib was added simultaneously with or after NRG1, the pathway network was still able to transmit signals even at a high lapatinib concentration of 1000 nM [48] (Fig. 2r, u and Supplementary Fig. S1g), suggesting that NRG1 is a factor that confers resistance to HER2-targeted therapies via reactivation of downstream signaling [53]. In addition, we showed that NRG1 through ligand-mediated receptor internalization and degradation can downregulate total HER3 level [48] (Fig. 2w) while lapatinib can upregulate total HER3 due to AKT-mediated feedback [48] (Fig. 2x). For pyrotinib which is less studied, we conducted in vitro experiments in SKBR3 cells to investigate its effect on cellular signaling (Fig. 3). Our experimental results, which were also quantitative reproduced by model simulations, suggest that pyrotinib can mediate dose-dependent inhibition of the ErbB network at both the receptor (Fig. 3a–c and Supplementary Fig. S1i) and intracellular levels (Fig. 3d, e and Supplementary Fig. S1j). In addition, pyrotinib treatment induces significant downregulation of total HER2 (Fig. 3f) but slightly induces HER3 (Fig. 3g), potentially due to the AKT-FOXO axis.

**Fig. 4:**
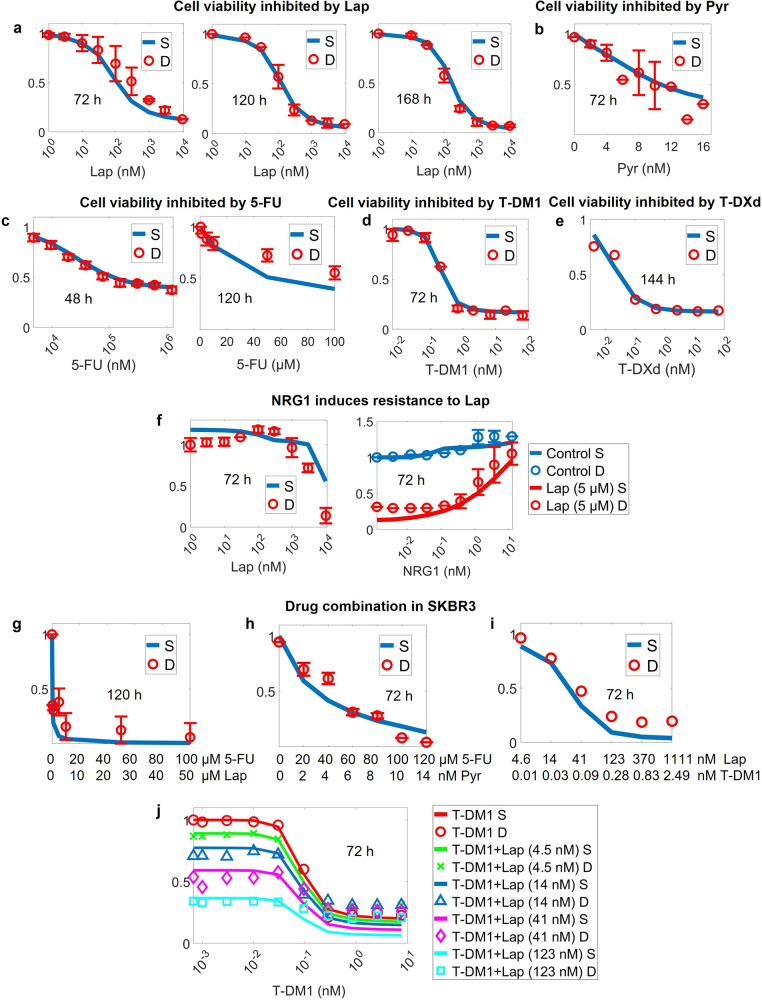
Model calibration of cell viability with single drug treatment data and validation with drug combinations. Dose-dependent inhibition of cell viability after exposure to (**a**) lapatinib for 72, 120, or 168 h, respectively, (**b**) pyrotinib for 72 h, (**c**) 5-FU for 48 or 120 h, respectively, (**d**) T-DM1 for 72 h and (**e**) T-DXd for 144 h. **f** Proliferation of cells treated with various concentrations of lapatinib for 72 h in the presence of NRG1 (left). The addition of NRG1 promotes cell proliferation and confers resistance to lapatinib dose-dependently (right). Dose-dependent inhibition of cell viability after combination treatments of (**g**) lapatinib with 5-FU for 120 h, (**h**) pyrotinib with 5-FU for 72 h and (**i**, **j**) lapatinib with T-DM1 for 72 h. All data are from experiments in the SKBR3 cell line. Y axes are relative viability levels (normalized to their respective DMSO controls, e.g., untreated conditions). S, simulation; D, experimental data.

Next, we explored the effects of T-DM1, T-DXd, lapatinib, pyrotinib and capecitabine on cell viability by linking signaling pathways to tumor cell growth (Fig. 4). Based on their mechanisms of action, lapatinib and pyrotinib indirectly regulate cell proliferation by reducing the pro-growth signals, while T-DM1, T-DXd and capecitabine (5-FU in in vitro assays) play direct roles in driving cell death. The model successfully captures all dose-response curves of the cell viability experimental data for the five drugs when treated alone [42, 48, 53–59] (Fig. 4a–e). Notably, growth/death parameters were estimated using only signaling and viability data from lapatinib (Fig. 4a), capecitabine/5-FU (Fig. 4c), T-DM1 (Fig. 4d) and T-DXd (Fig. 4e) groups, and still the model-predicted pyrotinib cell viability profile matches well with the observed experimental data (Fig. 4b) which provides a strong validation. Besides, both simulation and data show that the addition of NRG1 can significantly rescue the lapatinib-induced inhibition of SKBR3 cell viability [48, 53] (Fig. 4f), further confirming that NRG1 could be a cause of resistance to HER2-targeted therapies. We also performed the calibration procedure using data from other cell lines with different HER2 expression levels (medium and low, Supplementary Fig. S2). Model simulations also captured the experimental data quite well in another three cell lines (Supplementary Fig. S2a–k), indicating that our mechanistic cancer cell model framework can be generalized to study other HER2 expressing cells. From the dose-response profiles in these cell lines with lower HER2 expression, we also observe that HER2-targeted therapies are generally less potent.

In terms of model validation at the cell level, dose-response data for different drug combination regimens in SKBR3 were used (Fig. 4). These include lapatinib in combination with capecitabine [58] (Fig. 4g), pyrotinib in combination with capecitabine [55] (Fig. 4h), and lapatinib in combination with T-DM1 [60] (Fig. 4i, j), together with over 20 different dose combinations. Comparison between the quantitative experimental results and corresponding model simulations indicated that our model successfully predicts the synergistic effects of different drug combinations and different dose combinations on cell viability. Moreover, in another HER2 high expression cell line BT-474, the model was also successful in predicting the dose-response curves of lapatinib combined with T-DM1 (Supplementary Fig. S2l, m), again demonstrating the predictive power of our model.

### In vivo translation of the model enables accurate prediction of therapy-induced tumor growth inhibition

**Fig. 5:**
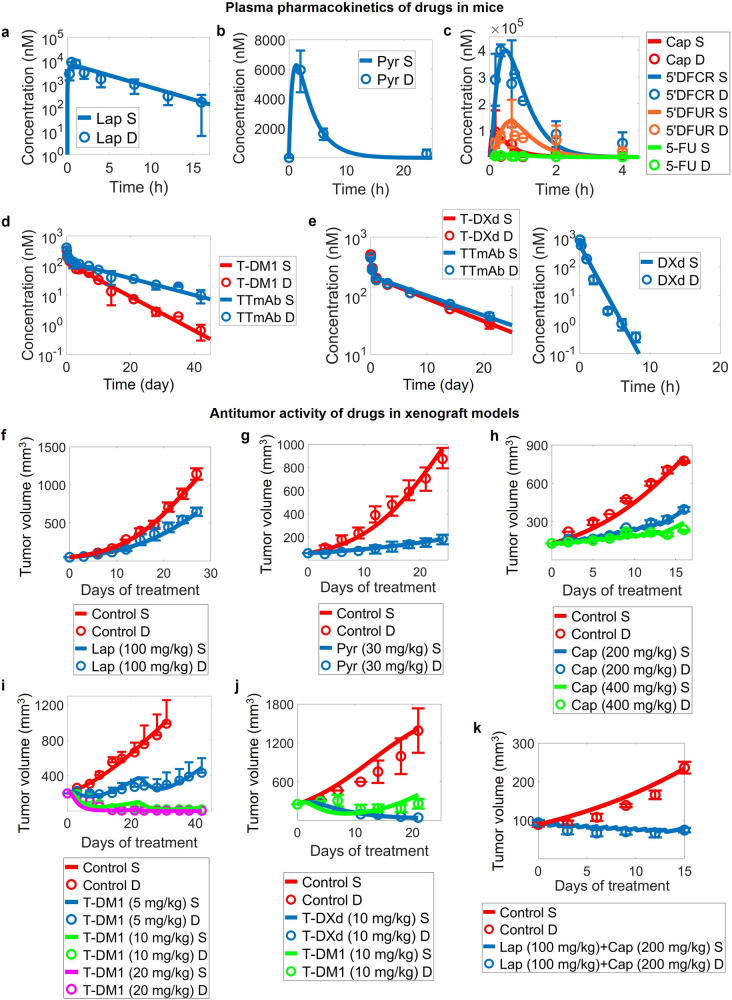
In vivo translation of the quantitative systems pharmacology model. Plasma pharmacokinetics of (**a**) lapatinib at 60 mg/kg, (**b**) pyrotinib at 80 mg/kg, (**c**) capecitabine at 755 mg/kg, (**d**) T-DM1 at 3 mg/kg, (**e**, left) T-DXd at 3 mg/kg and (**e**, right) DXd at 1 mg/kg in mice. In vivo antitumor activity of (**f**) lapatinib, (**g**) pyrotinib, (**h**) capecitabine, (**i**) T-DM1 and (**j**) T-DXd in breast cancer xenograft models. Tumor kinetics in (**f**, **g**) are from experiments in SKBR3 xenografts, in (**h**, **i**) are from KPL4 xenografts and in (**j**) are from a breast cancer PDX model with HER2 overexpression. **k** Model-predicted and in-house experimentally measured tumor growth kinetics in SKBR3 xenograft mice that received combination regimen of lapatinib plus capecitabine. In the simulations, tumors were allowed to grow to certain volumes before drug administration according to the different studies referenced and the maximum tumor volume was fixed to 2000 mm^3^. We assume that the weight of a mouse is approximately 20 g. S, simulation; D, experimental data.

Now that the model was able to quantitatively describe the biological behavior occurring within cells and predict drug effects on cell viability in vitro, we then further expand the cell-level model to describe and predict drug activity and tumor growth in vivo (Fig. 5). To achieve this translation, we assumed that all parameters related to signal transduction, drug-target inhibition and intracellular drug processing were held constant, while certain parameters controlling cell proliferation and death (e.g., umax, dmax, w14, w15) can differ between in vivo and in vitro scenarios given the significant changes of the cellular microenvironment. In addition, we formulated compartmental pharmacokinetic models for lapatinib, pyrotinib, capecitabine, T-DM1 and T-DXd, respectively [27–29] and we calibrated PK parameters using experimental data collected in mice. The PK modules accurately predicts plasma drug concentrations for all five therapeutics in mice [61–65] (Fig. 5a–e). Then, tumor growth kinetics in breast cancer xenografts treated with clinically relevant dosing regimens of lapatinib, pyrotinib, capecitabine, T-DM1 and T-DXd were used for model optimization and performance checking. Model-simulated tumor growth curves under lapatinib and pyrotinib treatment can simultaneously match the experimental data from HER2^+^ cell line-derived mouse xenografts (CDX) [66, 67] (Fig. 5f, g and Supplementary Fig. S3a, b). Similarly, for capecitabine and T-DM1, we used the control and lower dose drug-treated tumor growth curves as optimization datasets and then simulated in vivo tumor growth inhibition at the higher doses to evaluate the predictive capacity of the model. Resulting model simulations accurately characterized the dose-dependent and time-dependent inhibition of HER2^+^ tumors in CDX mice [68, 69] (Fig. 5h, i and Supplementary Fig. S3c), with T-DM1 being administered in two different regimens and five different doses in total. For T-DXd, we fitted the tumor growth kinetics in a breast cancer patient-derived xenograft (PDX) model with HER2 overexpression treated with T-DXd [42] (Fig. 5j). In this data, the T-DM1-treated tumor kinetics were also successfully reproduced at the same time using its respective parameters. To further validate the model in vivo, we measured tumor growth kinetics in mice under combination regimen of lapatinib plus capecitabine (experimental protocols are described in Methods). Compared to the control group, lapatinib plus capecitabine significantly slowed down tumor growth in mice bearing SKBR3-derived tumors (Supplementary Fig. S8a–c), as predicted by our model simulations (Fig. 5k). These results again demonstrate the important translational potential of our novel multiscale mechanistic model as an efficient computational platform for preclinical target evaluation and efficacy prediction.

### Sensitivity analyses enable identification of parameters that most significantly influences tumor growth and in silico generation of heterogeneous tumor response phenotypes

**Fig. 6:**
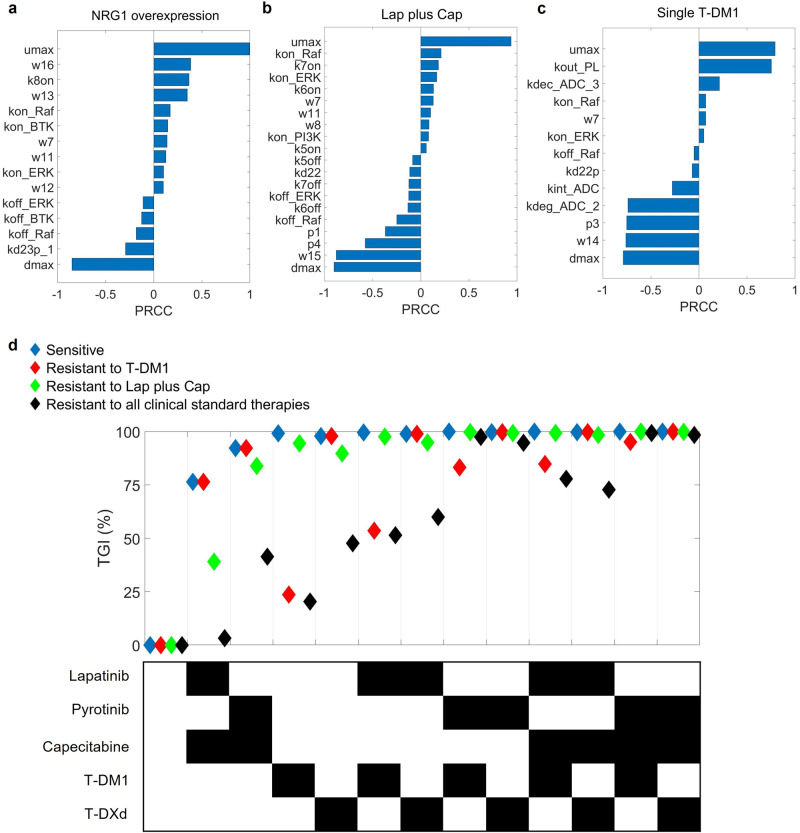
Sensitivity analysis and simulation of diverse treatment responses reflecting heterogeneous individual phenotypes. Partial rank correlation coefficients (PRCC) indices for parameters that significantly impact tumor volume (with absolute PRCC values greater than 0.05) under (**a**) NRG1 overexpression, (**b**) lapatinib plus capecitabine and (**c**) single agent T-DM1 conditions. The positive or negative signs of PRCC values represent a positive or negative effect on the model output. **d** Model simulations of tumor growth inhibition (TGI) after one cycle of treatment (e.g., TGI measured on day 20) using 13 different regimens for the four different response phenotypes, with blue representing a phenotype sensitive to all treatments, red—resistant to single T-DM1, green—resistant to lapatinib plus capecitabine, and black—resistant to all existing clinical standard therapies (lapatinib or pyrotinib plus capecitabine, single agent T-DM1 and T-DXd). The dosages are given as follows: lapatinib 100 mg/kg, qd; pyrotinib 30 mg/kg, qd; capecitabine 400 mg/kg, d1–d14 (e.g., days 1–14 of a 21-day cycle); T-DM1 30 mg/kg, q3w; T-DXd 10 mg/kg, q3w.

We then performed global sensitivity analyses using the PRCC method to identify parameters that most significantly affect model output (cell number or tumor volume) under different conditions (no external stimuli, NRG1 overexpression, lapatinib plus capecitabine, pyrotinib plus capecitabine, single agent T-DM1 and T-DXd). The overall trends in different scenarios were generally similar (Fig. 6a–c and Supplementary Fig. S4). As expected, the proliferation and death rates of tumor (umax and dmax) were the most critical parameters positively and negatively related to tumor volume, respectively. The activity of Raf/MAPK pathway (with relevant parameters including kon_Raf, koff_Raf, kon_ERK, koff_ERK, w7, w8 and w11) was another important factor affecting model output under all conditions. In the case of NRG1 overexpression, the binding rate of NRG1 to HER3 (k8on) was identified as a significant parameter that impacts tumor growth (Fig. 6a), indicating that this physiological process could be an important therapeutic target for NRG1-overexpressing tumors. In the presence of T-DM1 or T-DXd treatments, sensitivity analyses demonstrated that parameters that have substantial impacts on tumor volume are associated with the unique mechanism of action of HER2-ADC, such as the internalization rate of ADC (kint_ADC), the proteasomal degradation rate of ADC (kdeg_ADC_2), and the efflux or permeation rate of payloads (kout_PL, kper_PL) (Fig. 6c and Supplementary Fig. S4c). These parameters mechanistically correspond to several T-DM1 resistance mechanisms, including defective internalization [70], impaired lysosomal processing [71] and overexpression of drug efflux pumps [72, 73]. The above sensitivity results from PRCC also qualitatively agreed with those analyzed using the Sobol method (Supplementary Fig. S5). In addition, we performed model identifiability analysis using the bootstrapping method with a focus on the most influential parameters, and the results suggested relatively robust clustering of these parameters against our calibration datasets (Supplementary Fig. S6).

Since patients with HER2^+^ BC may respond differently to HER2-targeted therapies due to heterogeneity, we thus simulated four phenotypes with distinct response patterns to treatments by adjusting parameters based on the results of sensitivity analyses (Fig. 6d). Initially, the basic version of the model (representing a standard HER2^+^ CDX) was sensitive to all regimens (Fig. 6d, blue). By increasing the efflux rate of DM1 catabolites (kout_PL), we simulated the T-DM1-resistant phenotype (Fig. 6d, red), which remained effective against T-DXd and therapies containing TKIs probably because of their distinct mechanisms of action. By increasing both the proliferation rate of tumor (umax) and the activation rate of Raf (kon_Raf) thereby increasing tumor growth and mimicking the aberrant transduction of Raf/MAPK signaling, we modeled the lapatinib-resistant phenotype (Fig. 6d, green). However, it was still sensitive to pyrotinib plus capecitabine and new combination strategies such as TKI plus ADC, triple combination of TKI, capecitabine and ADC, according to the simulation results. We further increased the proliferation rate (umax) and were able to simulate a phenotype that was refractory to all clinically available treatments including lapatinib or pyrotinib plus capecitabine as well as single T-DM1 or T-DXd (Fig. 6d, black). We found that in this phenotype triple combinations of lapatinib, capecitabine and ADC (10th and 11th columns) could partially inhibit tumor growth, while double combinations of pyrotinib plus ADC (8th and 9th columns) have significantly better efficacy. These results suggested that with minimal physiological parameter tuning, the model was able to simulate heterogeneous HER2^+^ breast cancer phenotypes with differential drug response profiles, thus allowing explainable in silico exploration of different drug combinations to deal with various resistance mechanisms during HER2^+^ mBC treatments.

### Simulations provide mechanistic insights into treatment sequencing and new combination strategies for improving efficacy and targeting resistance

**Fig. 7:**
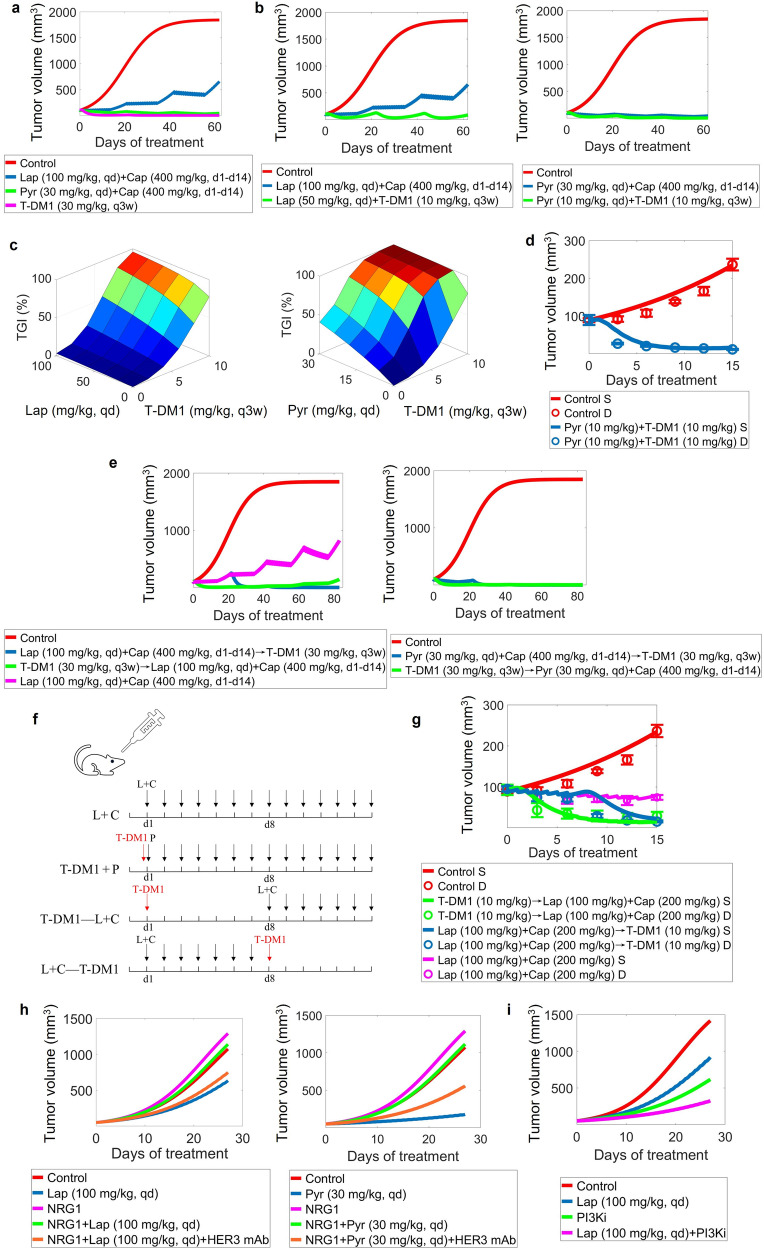
Model evaluation and experimental validation of tumor response kinetics in mice under different drug treatment strategies. **a** Simulated efficacy of different drug regimens used in clinical practice, including lapatinib plus capecitabine, pyrotinib plus capecitabine and single T-DM1. **b** Simulated antitumor effects of the new combination regimen of lapatinib or pyrotinib plus T-DM1 versus classic lapatinib or pyrotinib plus capecitabine. **c** Simulated tumor growth inhibition (TGI) in response to combinations of lapatinib or pyrotinib with T-DM1 over a range of doses (lapatinib 20–100 mg/kg qd, pyrotinib 6–30 mg/kg qd, and T-DM1 2–10 mg/kg q3w, respectively). Tumor volumes were analyzed after three treatment cycles (e.g., on day 62). **d** QSP model prediction and in-house experimental validation of tumor growth kinetics in vivo in response to combination regimen of pyrotinib (10 mg/kg) plus T-DM1 (10 mg/kg); S, simulation; D, experimental data. **e** Simulated antitumor effects of sequential therapies of lapatinib or pyrotinib plus capecitabine followed by T-DM1 or T-DM1 followed by lapatinib or pyrotinib plus capecitabine. **f** The drug administration protocol of the in vivo mouse xenograft experiments (L, lapatinib; C, capecitabine; P, pyrotinib; see Methods section for details). **g** QSP model prediction and in-house experimental validation of tumor growth kinetics in vivo in response to sequential regimen between T-DM1 and lapatinib plus capecitabine; S, simulation; D, experimental data. **h** Simulated tumor growth trends under single lapatinib or pyrotinib, NRG1 (representing the overexpression-induced resistant scenario), and the combination as well as in the presence of HER3 mAb. **i** Simulated tumor growth trends under single lapatinib, PI3K inhibitor and their combination when tumor growth is highly dependent on the PI3K/AKT pathway. See the legends for the detailed dosage and frequency of administrations, where a treatment cycle is 21 days and d1–d14 means capecitabine is administered on days 1–14 of each cycle.

Using our mechanistic QSP model, we then explored different drug treatment strategies and predicted their preclinical efficacy to provide insights for future clinical investigations. We were primarily interested in the second-line treatments for HER2^+^ mBC (e.g., TKI plus capecitabine, HER2-ADC) as well as the potential of new targets, new regimens, and new combinations. Clinical trials have been conducted to compare the efficacy of lapatinib plus capecitabine versus pyrotinib plus capecitabine [6], and lapatinib plus capecitabine versus T-DM1 [74]. However, prior studies have not elucidated the comparative efficacy of pyrotinib plus capecitabine versus T-DM1, so we investigated this question using our model. Interestingly, the two above treatment strategies induced very similar tumor regression at the preclinical level according to our simulations (Fig. 7a), and the tumor inhibitory potency of both strategies was superior than that of lapatinib plus capecitabine which is consistent with clinical trial results [6, 74]. For TKIs, we further showed that the combination of lapatinib or pyrotinib with T-DM1 or T-DXd (even at significantly lower doses) appeared to be more efficacious than TKIs plus capecitabine (Fig. 7b and Supplementary Fig. S7a). We systematically explored the antitumor response to such new combinations at different dose pairs, and the predicted dose-response surfaces indicate synergistic effects of both TKIs in combination with T-DM1 according to the Bliss independence principle. Particularly for pyrotinib, sufficient inhibition (~80%) of tumor growth can be achieved at markedly reduced dosages of pyrotinib (6 mg/kg) plus T-DM1 (6 mg/kg), compared to the regular single-agent dosages. To achieve this similar level of tumor inhibition, relatively higher doses of combined lapatinib and T-DM1 were required (80% inhibition under lapatinib 80 mg/kg plus T-DM1 8 mg/kg) as expected (Fig. 7c). We further validated the anti-tumor efficacy of pyrotinib plus T-DM1 in SKBR3 xenograft mice: as quantitatively predicted by our QSP model, such a combination already exhibits a very strong eradicative effect on tumor growth in vivo (Fig. 7d and Supplementary Fig. S8a–c), which is comparable or even superior than single agents given at significantly higher doses. For T-DXd, its combination with TKIs also showed more potent efficacy and can achieve significant tumor growth inhibition at reduced doses (Supplementary Fig. S7b). A comparison of model-predicted TGI and Bliss-predicted TGI supporting the synergism of these four combinations was provided in Supplementary Table S2.

Treatment sequencing is another heterogenous factor that can impact individual clinical outcome. For HER2^+^ mBC, we applied our model to investigate the potential impact of different second-line treatment sequences, considering the TKI-containing regimens and single agent T-DM1 or T-DXd. We simulated preclinical tumor growth kinetics under the following two conditions: TKI plus capecitabine for one cycle followed by ADC or ADC for one cycle followed by TKI plus capecitabine. Our model simulations suggest that lapatinib plus capecitabine followed by T-DM1 could induce durable tumor regression, while T-DM1 followed by lapatinib plus capecitabine appears to be more potent in the short term and is equally effective in the long run; in terms of tumor growth inhibition, both strategies are more effective than the conventional regimen of lapatinib plus capecitabine alone (Fig. 7e). This comparative phenomenon was also quantitatively validated by our in-house experiments that tested the anti-tumor effect of sequential dosing regimens in mice (Fig. 7g and Supplementary Fig. S8d–f, experimental protocols are shown in Fig. 7f), confirming the predictive capacity of our QSP model. For pyrotinib, both therapy sequences (pyrotinib plus capecitabine first then T-DM1 or T-DM1 first then pyrotinib plus capecitabine) had similar and extraordinary effects in eliminating tumor growth (Fig. 7e). For T-DXd, due to its strong tumoricidal potency, treatment ordering of T-DXd versus both TKIs plus capecitabine can always induce durable tumor regression regardless of sequencing (Supplementary Fig. S7c).

Drug resistance in HER2^+^ mBC is also of significant clinical importance. A potential factor for resistance identified from our previous model analysis is the presence of high NRG1, while in patients it is an oncogenic feature observed across multiple cancer types [75, 76]. In the NRG1-high expression scenario, our model predicts that it greatly diminishes the TKI-mediated tumor growth inhibition (Fig. 7h). The simulated tumor growth trends were in accordance with the experimental results by Nonagase et al. [53] where they find significantly less tumor reduction in response to lapatinib in a NRG1-expressing CDX model. To explore therapeutic strategies targeting this resistance mechanism, our simulations showed that blocking the binding between NRG1 and HER3 (mimicking the effect of HER3 antibodies, as suggested by the sensitivity analysis) can overcome resistance and restore the antitumor activity of TKI (Fig. 7h), suggesting a potentially new route for translational research in HER2^+^ and NRG1^+^ /HER3^+^ patients. Another factor that confers lapatinib resistance is the aberrant activation of PI3K/AKT pathway, which was represented in our model as a much higher dependency on PI3K signaling for tumor growth. Simulated results indicated continued tumor growth under lapatinib alone but significantly reduced growth when we decrease the activation rate of PI3K (mimicking the effect of PI3K inhibitor) (Fig. 7i), and this is consistent with the experimental findings from a HCC1954 cell xenograft model with endogenous PIK3CA mutation [77]. In addition to these primary resistance scenarios, our model framework can also be used to simulate progressive acquired resistance, as demonstrated in Supplementary Fig. S7d.

## Discussion

HER2^+^ BC is known for its rapid progression and aggressiveness, and patients are more prone to tumor metastasis and relapse [2]. Despite the availability of various HER2-targeting drugs, optimizing treatment strategy and developing new agents for patients with advanced disease (e.g., HER2^+^ mBC) are of significant research and clinical values. Given the time and money costs of clinical trials, developing new methods to accurately and prospectively assess the clinical translational value of different candidate therapies is an essential challenge. In this work, we have constructed a novel mechanistic quantitative systems pharmacology model describing the underlying pathophysiological processes of HER2+ BC, from ligand-receptor binding to downstream signaling and finally to tumor growth, while incorporating the distinct modalities and mechanisms of various state-of-the-art therapeutics. We used a large variety of in vitro and in vivo experimental data during model calibration and validation, achieving a quantitative and accurate description of cellular signaling, time-response, dose-response, and tumor growth kinetics. This further allowed us to probe into the efficacy of various therapeutic strategies at the preclinical level and suggested important directions for future translational drug research. When performing in vitro to in vivo translation, a general assumption is that the biochemical network structure and parameter values are largely conserved, while the microenvironment could be different (so are the related parameters). This has led to smaller values of growth and death parameters under in vivo versus in vitro conditions, a pattern that is also suggested by several other modeling studies [78, 79]. As HER2^+^ BC is a highly complex and heterogenous disease, we envision our model could serve as an evolving in silico platform that could be constantly refined and expanded by adding new mechanistic details such as new drug-acting pathways and resistance mechanisms. For example, at the population level, double positive of HER2 and estrogen receptors (ER) occurs in approximately 50% of HER2^+^ BC patients [80], and 20% patients have cyclin E overexpression [80]. Future model expansions around these pathophysiological features would help us better understand the tumorigenesis and therapeutic outcome of such particular patient subgroups in order to advance the practice of personalized medicine in HER2^+^ BC.

Using this model, we performed a series of efficacy analyses to provide insights for clinical research. First, we compared the efficacy of pyrotinib plus capecitabine with T-DM1 since no published study (animal or human) has yet compared these two regimens head-to-head. The model predicted that both regimens were notably effective in inhibiting tumor growth in mouse, with T-DM1 being slightly more potent than pyrotinib plus capecitabine (Fig. 7a). If we compare the PHOEBE study (pyrotinib plus capecitabine) with the EMILIA study (T-DM1) as the control arms in both studies received the same regimen (lapatinib plus capecitabine), we would see that for efficacy, T-DM1 in the EMILIA study significantly prolonged overall survival (OS) in the Asian population by nearly one year and reduced the risk of death by 57.2% [81], whereas pyrotinib plus capecitabine in the PHOEBE study also had an OS benefit but not as much as that of T-DM1 in Asians, and there was no OS benefit in the trastuzumab-resistant population [82]. Although authors of the PHOEBE study, through preliminary cross-trial analyses, proposed that the efficacy of pyrotinib plus capecitabine would be similar to T-DM1, more reliable studies are still needed if one wants to make a direct comparison between these two regimens [6]. Secondly, we tested a novel combination of TKI plus ADC that has never been used in clinical practice, since their mechanisms of action and resistance differ from each other which theoretically provides an opportunity for combination. The model predicted that such combinations were able to induce durable tumor regression in mice even when the doses were significantly reduced (Fig. 7b and Supplementary Fig. S7a), which potentially suggests less toxicity issues. Model-based dose-response analysis recommended pyrotinib plus ADC as the preferred option compared with lapatinib plus ADC, as it exhibited stronger synergism at lower doses (Fig. 7c and Supplementary Fig. S7b). Supporting our predictions, a phase Ib study testing T-DM1 plus lapatinib and nab-paclitaxel in HER2^+^ mBC has achieved excellent results: patients had relatively well tolerability with a high objective response rate (ORR) of 85.7% (12/14) [83]. Another study of T-DM1 plus neratinib (a multi-target TKI) exhibited favorable antitumor activity as well: 12 of 19 (63%) evaluable patients had an objective response [20] and this was markedly higher than T-DM1 alone (44% in EMILIA). The third clinically-relevant question we were interested in is the optimal treatment sequence. Interesting, our model predicts that treating mice with only one cycle of T-DM1 followed by daily lapatinib plus capecitabine would still generate superior efficacy than direct lapatinib plus capecitabine (Fig. 7e). This suggested that longer disease stabilization and response duration may be achieved even if limited cycles of T-DM1 is received prior to lapatinib regimen, which has therapeutic implications for the portion of patients who have to discontinue T-DM1 as a result of adverse events (e.g., thrombocytopenia) in the real-world setting (occurring in 10% of 490 patients in the EMILIA study [5]).

The model identified NRG1 as a cause of TKI resistance in HER2^+^ BC due to its ability to bind to HER3 and HER4 and continuously activate downstream MAPK and AKT pathways. Focusing on the microenvironmental signals, Watson et al. identified NRG1 to reduce TKI efficacy in SKBR3 cell line and the addition of pertuzumab could reverse it as it blocks ligand-induced HER2/HER3 dimerization [84]. In fact, NRG1 fusions are considered as oncogenic drivers, occurring in 0.2% of all solid tumors and about 0.2% in breast cancers [75]. On the other hand, the incidence of HER3 overexpression in patients is much higher (30% in breast cancer [85]) and is associated with a poor prognosis [86]. Sensitivity analysis under the condition of NRG1 overexpression and our simulations suggested that HER3-targeted agents or HER3-containing combination therapy may be a promising direction in NRG1 fusion-driven or HER3-overexpressed tumors. Many pharmaceutical companies have already marched into the development of HER3-targeted drugs, of which a representative first-generation therapeutic being the anti-HER3 mAb seribantumab [87]. In a recent phase II study of seribantumab in patients with NRG1 fusion-positive advanced solid tumors (CRESTONE), preliminary results were presented with an ORR of 33% and a disease control rate of 92% in cohort 1 [88]. Other drugs such as zenocutuzumab (a HER2/HER3 bispecific antibody for NRG1 fusion-related tumors) and U3-1402 (a HER3-ADC that primarily targets HER3-expressing cells) have also announced encouraging results lately [89, 90]. In addition, a preclinical study revealed that inhibition of HER3 expression combined with lapatinib reduced MMTV-Neu mice tumor growth to a greater extent than either HER3 inhibition or lapatinib alone [91], suggesting the potential of HER3-containing combination regimens in treating HER2-overexpressing breast cancers.

Our mechanistic model is a solid example of model-driven in vitro to in vivo translation, particularly for efficacy evaluation and combination assessment, and it can be further advanced to the clinical level by enabling virtual patient generation and virtual clinical trials to predict the clinical efficacy and outcome of new therapies in breast cancer patients. A number of studies have demonstrated the feasibility and value of such a mechanistic model-based translational strategy in complex human diseases [92–94]. In expediting preclinical to clinical translation, Qiao et al. developed a QSP model of immuno-oncology to understand the variability in tumor kinetics in syngenetic mice treated with anti-CTLA4 antibody and proposed a combination regimen of anti-CTLA4 with therapeutics that expand CD8^+^ T cells for non-responding tumors [95]. Another case in point is a computational model of the MAPK signaling network developed by Kirouac et al. to predict the clinical outcomes of ERK inhibition in colorectal cancer with the BRAF-V600E mutation [78], in which the authors started from in vitro cell culture data to in vivo animal response to clinical prediction using one general model framework. Another recent example is Zhu et al. that constructed a translational PK/PD model using data from in vitro tumor organoids and established translational scaling of the model to predict the clinical response of oxaliplatin and irinotecan in colorectal cancer [96]. Therefore, an immediate next step is to extend our model to the clinical level and make full use of the multimodal data from published trials on HER2^+^ mBC (e.g., individual data from spider plots and waterfall plots, population-level ORR, PFS of different regimens) as well as original biomarker data from in-house patient samples to collectively generate a plausible and accurate virtual patient population. To better advance this modeling framework to the clinical level, potential drug-induced toxicities can also be incorporated through modeling methods. For example, for HER2-ADC, mechanism-based models were developed to predict platelet reduction after T-DM1 treatment [97, 98]; for TKIs which may cause cardiotoxicity, a recent model relating drug-induced perturbations of myocardial contractility to clinical ejection fraction can serve as a modeling basis [99]. Furthermore, as we (previously with Johns Hopkins collaborators) have already developed a comprehensive quantitative systems pharmacology platform for immuno-oncology (IO) [100, 101], adding an immune module to our breast cancer model to enable the simulation of various modalities of immunotherapy in combination with HER2-targeted therapies is also of great importance, given the competitive drug development landscape of HER2^+^ mBC and many ongoing trials in this field [7].

### Limitations of the study

As our model calibration and validation included data from different literature studies, we did make a unifying assumption that all data come from similar and comparable cell culture conditions if the experiments used the same cell lines (different doses were accounted explicitly and numerically), despite the potential presence of other technical residuals that could differ between labs. According to the results, the same model structure and the same set of parameters performed well in describing all the data from the SKBR3 cell line simultaneously, which confirmed the accuracy and generality of the model. Still, future efforts should pay attention to these experimental factors and protocol details when modeling in vitro cell cultures. Another limitation worth ongoing attention is possible drug-drug interactions (DDI) at the PK level when simulating drug combinations. Within our model scope, although no clear DDI evidence was found so far regarding coadministration of TKI plus capecitabine or TKI plus ADC in the literature [102, 103], it always remains an important issue for the clinic as new molecular entities are continuously being developed. Finally, in order to characterize drug resistance, here we simulated primary resistance by enforcing NRG1 overexpression or high PI3K dependency, and acquired resistance by assuming that cumulative drug exposure could induce reduction in drug potency. Other approaches may also be utilized here to define resistance such as the evolution-based method, as demonstrated in a recent modeling work where the authors explicitly included a sensitive subclone and a cascade of resistant subclones of tumor cells and allowed transition between subclones to physically reflect the resistance acquiring process [104]. Therefore, in the future new mechanisms and methodologies could be introduced into our model to better address resistance from the molecular and spatiotemporal aspects.

### Supplementary information

Supplementary Materials

Supplementary Table S1

Supplementary Table S1 legend
