# Supplementary material for: Quantitative systems pharmacology modeling of HER2-positive metastatic breast cancer for translational efficacy evaluation and combination assessment across therapeutic modalities
Source: Acta Pharmacol Sin. 2024 Feb 15;45(6):1287–304. doi: 10.1038/s41401-024-01232-9 (PMC11130324; doi:10.1038/s41401-024-01232-9)
Supplement: Supplementary file 1 — Supplementary Materials [file 41401_2024_1232_MOESM1_ESM.docx]

**Supplementary Materials**

**Quantitative systems pharmacology modeling of HER2-positive metastatic breast cancer for translational efficacy evaluation and combination assessment across therapeutic modalities**

Ya-ting Zhou, Jia-hui Chu, Shu-han Zhao, Ge-li Li, Zi-yi Fu, Su-jie Zhang, Xue-hu Gao, Wen Ma, Kai Shen, Yuan Gao, Wei Li, Yong-mei Yin, Chen Zhao

**This file includes:**

**Supplementary Fig. S1** Additional model calibration of phospho-receptors and downstream PI3K/AKT, Ras/MAPK signal transduction. See also Fig. 2.

**Supplementary Fig. S2** Additional model calibration of cell viability under single drug treatment in other HER2-expressing cell lines and model validation of drug combination efficacy in BT-474 cells. See also Fig. 4.

**Supplementary Fig. S3** In vivo translation of the QSP model in BT-474 xenografts as a validation. See also Fig. 5.

**Supplementary Fig. S4** Global parameter sensitivity analysis under three other in vivo conditions using the PRCC method. See also Fig. 6.

**Supplementary Fig. S5** Global sensitivity analysis using the Sobol method. See also Fig. 6.

**Supplementary Fig. S6** Parameter estimate distributions after bootstrapping.

**Supplementary Fig. S7** Additional model evaluations of tumor response kinetics in vivo under different treatment strategies. See also Fig. 7.

**Supplementary Fig. S8** In vivo experimental validation of model-predicted tumor growth kinetics in response to combination or sequential therapies. See also Fig. 7.

**Supplementary Fig. S9** Detailed model diagram. See also Fig. 1.

**Supplementary Table S2.** Comparison of model-predicted TGI and Bliss-predicted TGI of four drug combination regimens.


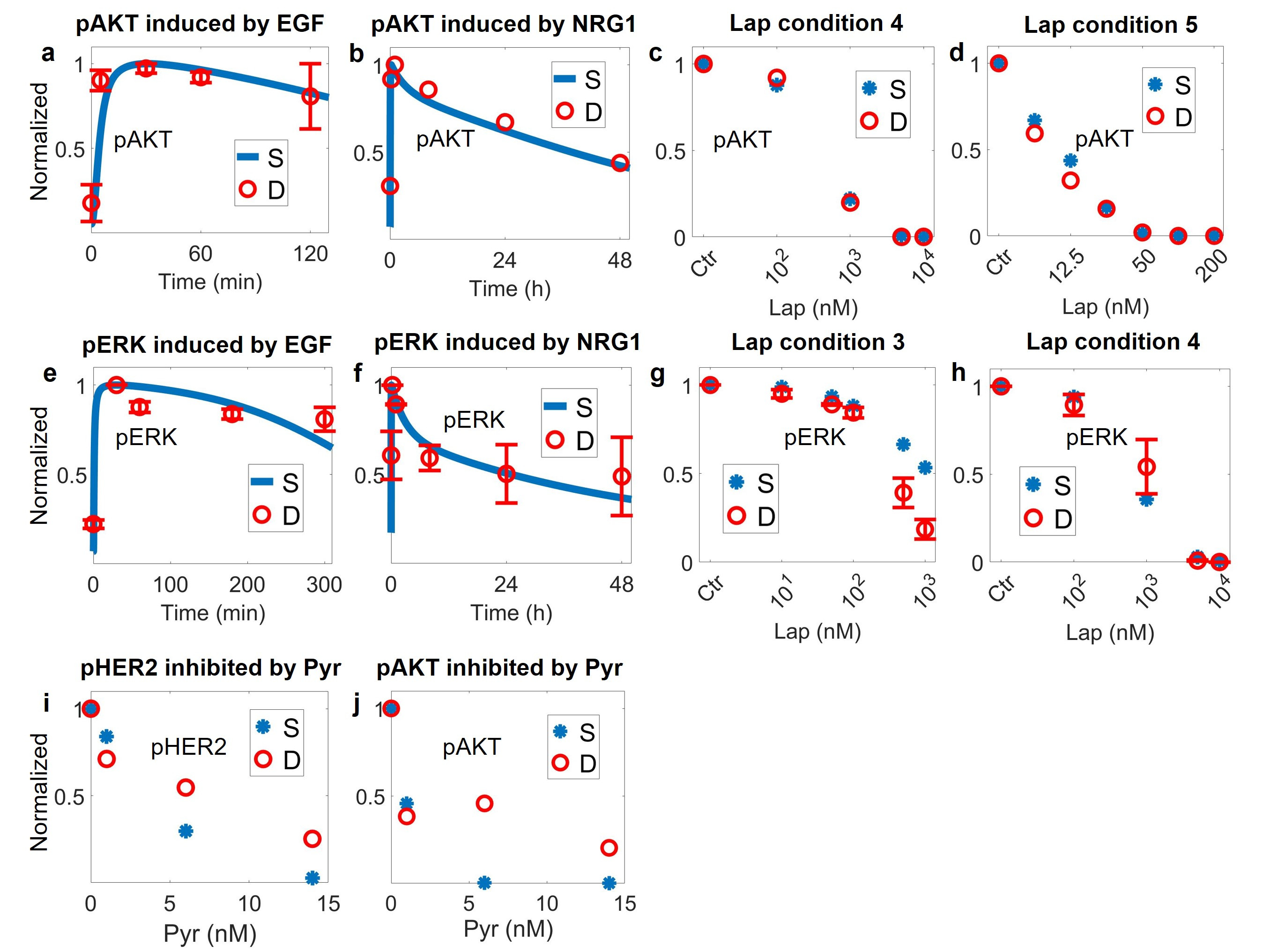


**Supplementary Fig. S1 Additional model calibration of phospho-receptors and downstream PI3K/AKT, Ras/MAPK signal transduction. See also Fig. 2. a** EGF (50 ng/mL) induces activation of AKT (Selitrennik et al.^[1]^). **b** NRG1 (50 ng/mL) induces activation of AKT (Novotny et al.^[2]^). **c, d** Lapatinib induces dose-dependent inhibition of AKT (Li et al.^[3]^, Amin et al.^[4]^). **e** EGF (100 ng/mL) induces activation of ERK (Chen et al.^[5]^). **f** NRG1 (50 ng/mL) induces activation of ERK (Novotny et al.^[2]^). **g, h** Lapatinib induces dose-dependent inhibition of ERK (Novotny et al.^[2]^, Li et al.^[3]^). **i, j** Pyrotinib induces dose-dependent inhibition of HER2 and AKT (Zhang et al.^[6]^, Yi et al.^[7]^). All data are from experiments in the SKBR3 cell line. Y axes are relative expression levels (normalized to their respective maximum values). Lap condition 3, NRG1 (50 ng/mL) for 15 min followed by lapatinib for 15 min; Lap condition 4, lapatinib for 3 h followed by EGF (100 ng/mL) for 15 min; Lap condition 5, lapatinib alone for 60 min; S, simulation; D, experimental data; Ctr, control/untreated condition.

**
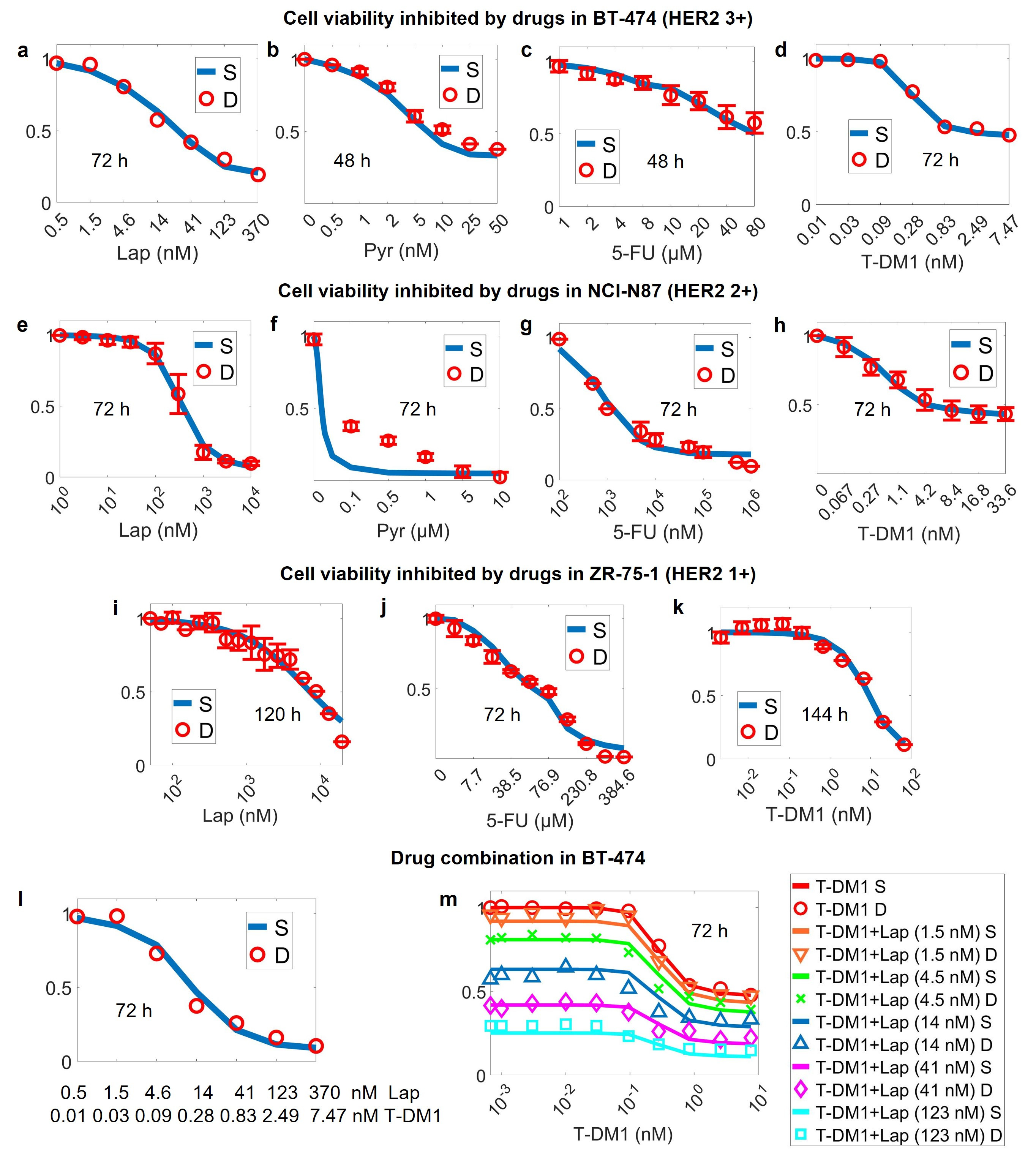
**

**Supplementary Fig. S2 Additional model calibration of cell viability under single drug treatment in other HER2-expressing cell lines and model validation of drug combination efficacy in BT-474 cells. See also Fig. 4. a**–**d** Dose-dependent inhibition of cell viability in the BT-474 cell line after exposure to **(a)** lapatinib for 72 h (Berry et al.^[8]^), **(b)** pyrotinib for 48 h (Bu et al.^[9]^), **(c)** 5-FU for 48 h (Yin et al.^[10]^), and **(d)** T-DM1 for 72 h (Berry et al.^[8]^), respectively. **e**–**h** Dose-dependent inhibition of cell viability in the NCI-N87 cell line after treatment with **(e)** lapatinib (Nonagase et al.^[11]^), **(f)** pyrotinib (Su et al.^[12]^), **(g)** 5-FU (Nakamura et al.^[13]^), and **(h)** T-DM1 (Zhang et al.^[14]^) for 72 h. **i**–**k** Dose-dependent inhibition of cell viability in the ZR-75-1 cell line treated with **(i)** lapatinib for 120 h (van Agthoven et al.^[15]^), **(j)** 5-FU for 72 h (Tsao et al.^[16]^), and **(k)** T-DM1 for 144 h (van der Lee et al.^[17]^), respectively. **l, m** Dose-dependent inhibition of cell viability after combinatory treatments of lapatinib with T-DM1 for 72 h (Berry et al.^[8]^). Y axes are relative levels (normalized to their respective DMSO controls, i.e., untreated conditions). S, simulation; D, experimental data.


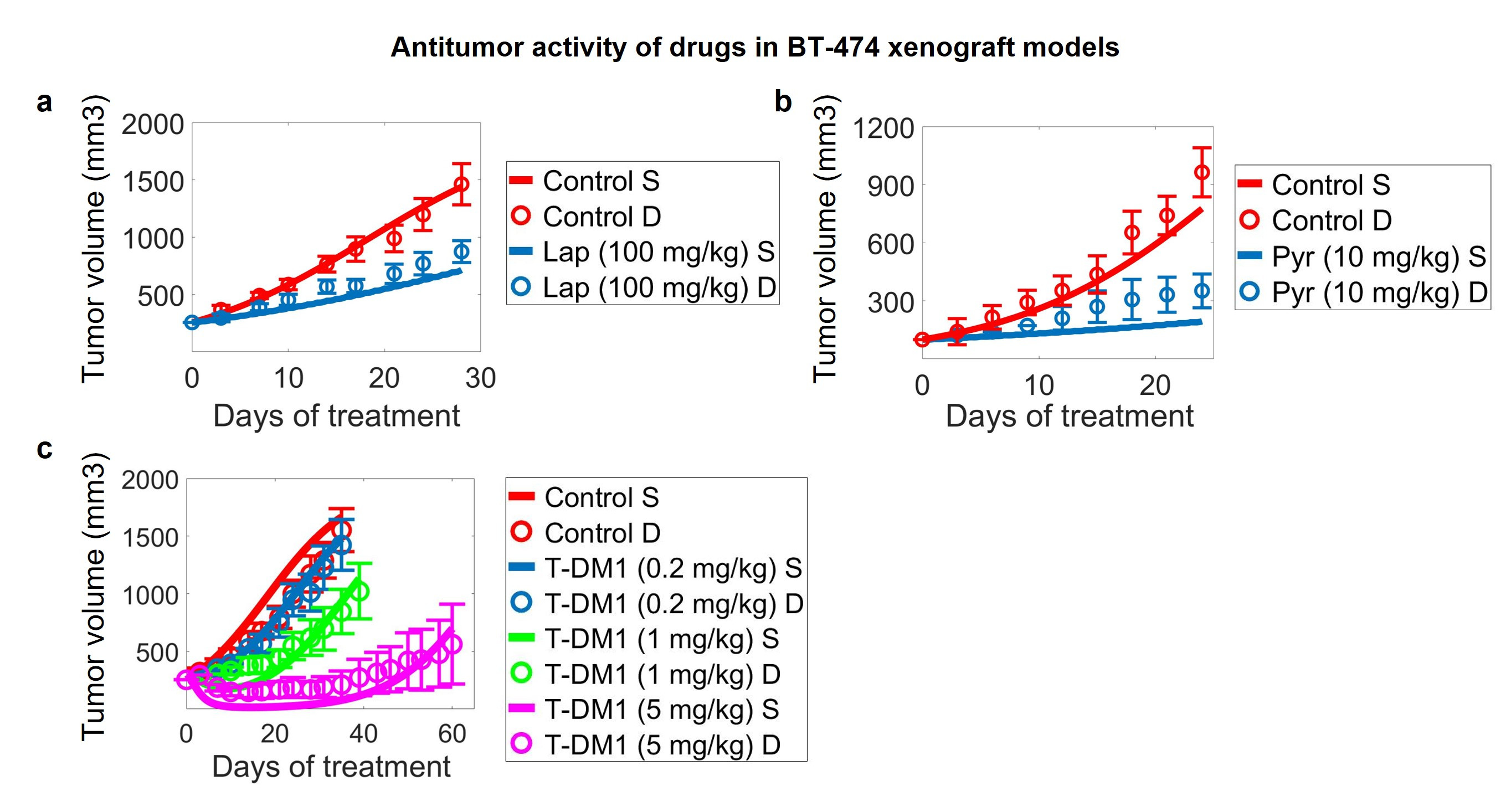


**Supplementary Fig. S3 In vivo translation of the QSP model in BT-474 xenografts as a validation. See also Fig. 5.** In vivo antitumor activity of **(a)** lapatinib (Chakrabarty et al.^[18]^), **(b)** pyrotinib (Zhang et al.^[6]^) and **(c)** T-DM1 (van der Lee et al.^[17]^) in BT-474 xenografts, respectively. In the simulations, tumors were allowed to grow to certain volumes before drug administration according to the different studies referenced and the maximum tumor volume was fixed to 2000 mm^3^. We assume that the weight of a mouse is approximately 20 grams. S, simulation; D, experimental data.

**
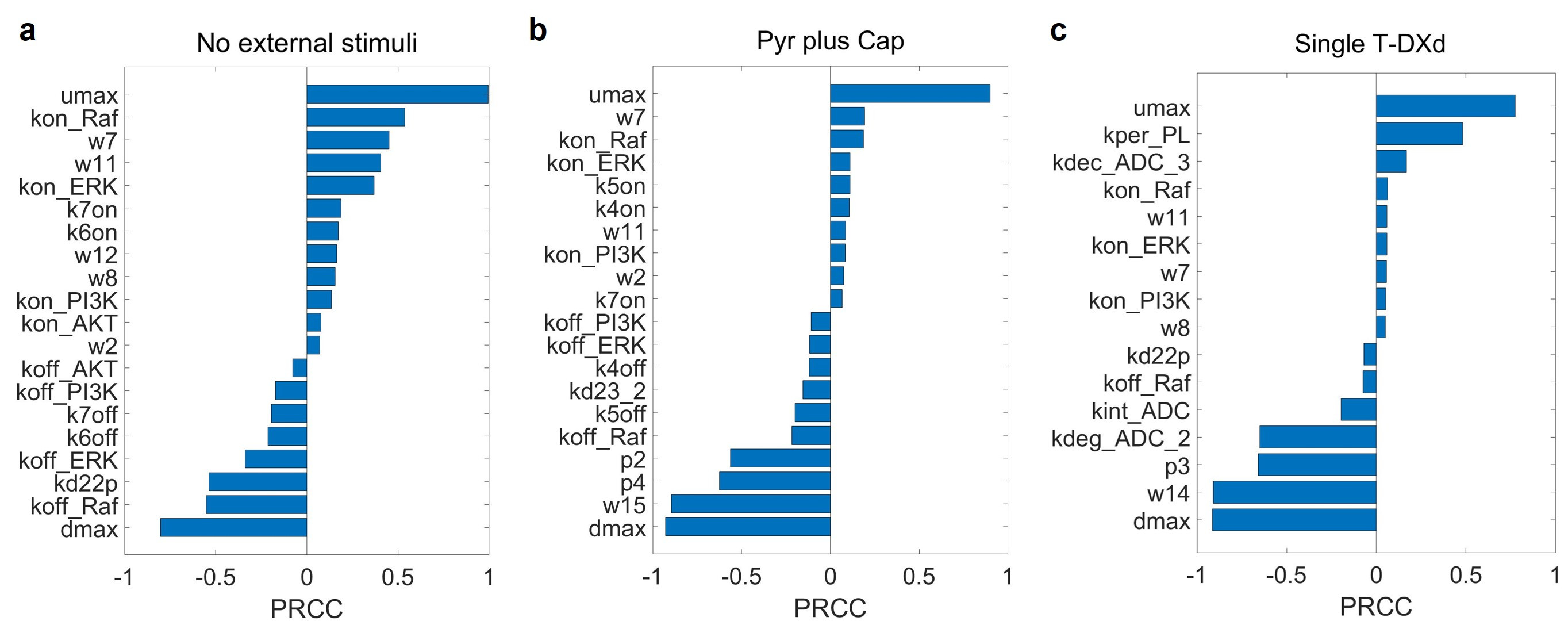
**

**Supplementary Fig. S4 Global parameter sensitivity analysis under three other in vivo conditions using the PRCC method. See also Fig. 6.** Partial rank correlation coefficients (PRCC) for parameters significantly affect tumor volume (with absolute PRCC values greater than 0.05) under **(a)** no external stimuli, **(b)** pyrotinib plus capecitabine and **(c)** single agent T-DXd conditions.

**
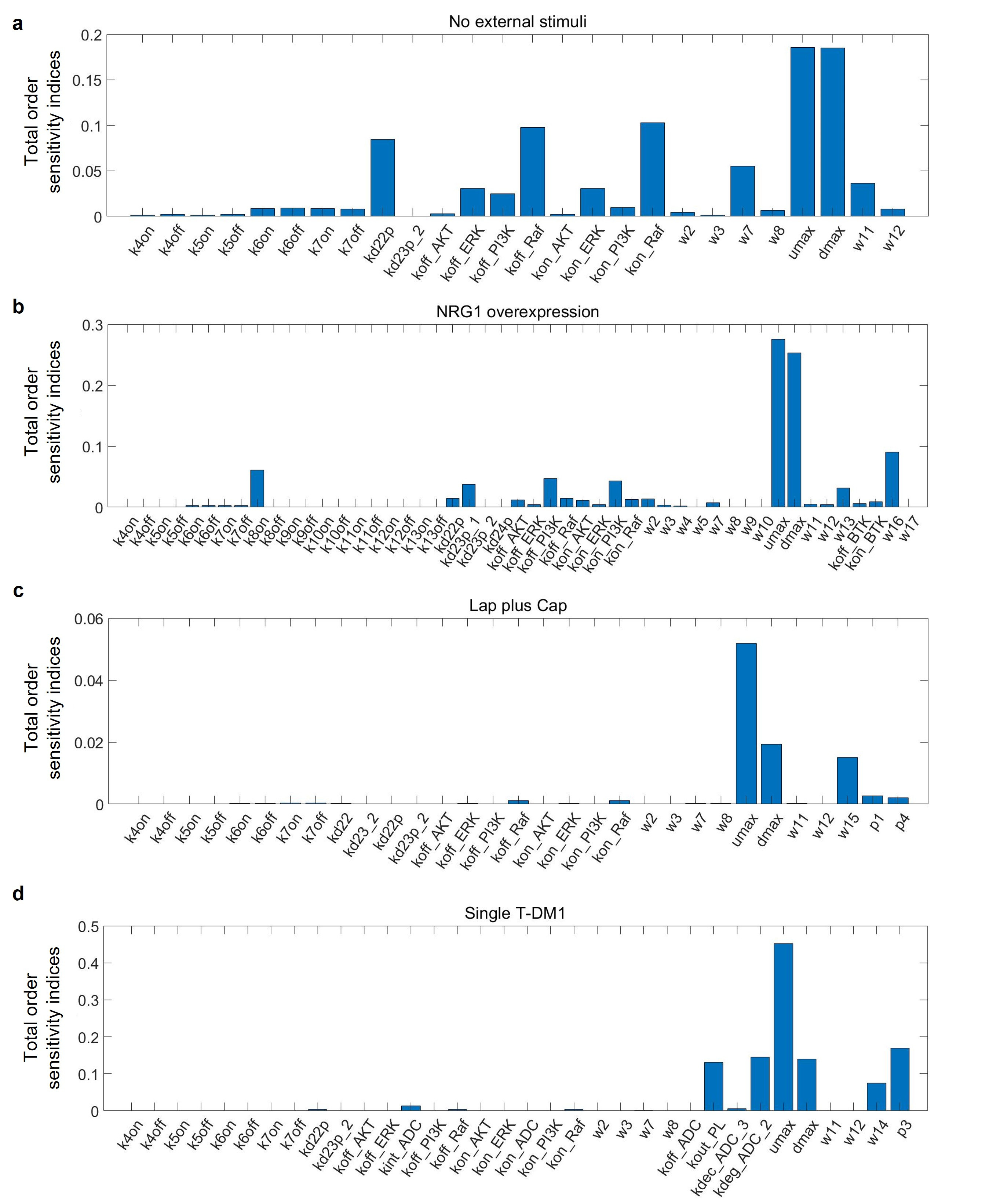
**

**Supplementary Fig. S5 Global sensitivity analysis using the Sobol method. See also Fig. 6.** Total-order sensitivity indices of parameters under **(a)** no external stimuli**, (b)** NRG1 overexpression, **(c)** lapatinib plus capecitabine and **(d)** single agent T-DM1 conditions. The greater the sensitivity indices are, the more crucial parameters are for the model.

**
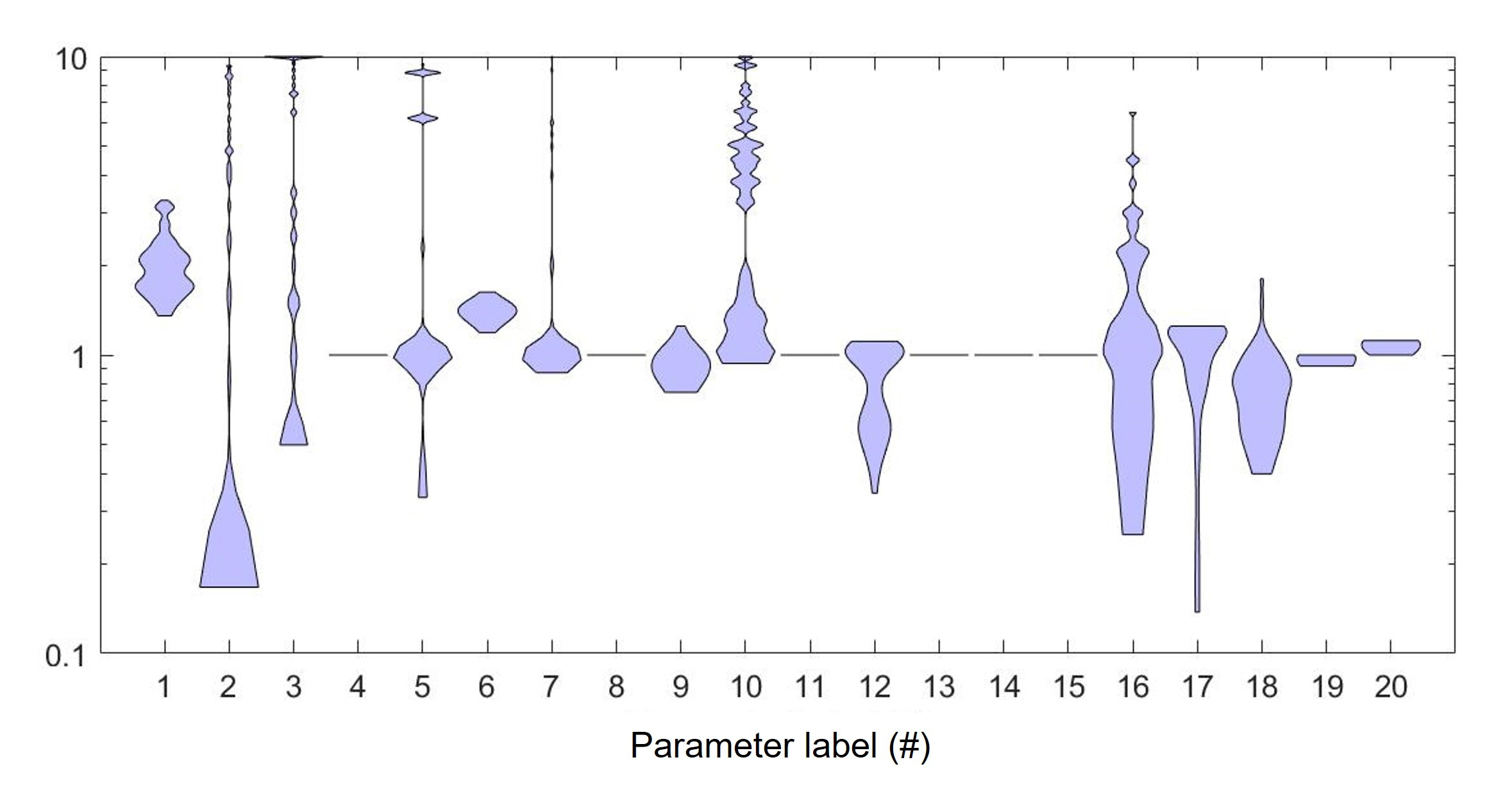
**

**Supplementary Fig. S6 Parameter estimate distributions after bootstrapping.** Parameter estimate distributions (represented by violin plots) of the top-20 most sensitive parameters. All parameter values were allowed to vary from 0.1× to 10× during bootstrapping and are normalized to their respective reference values for display (y-axis in log scale). The parameters labeled #1–20 are k8on, k9on, k9off, k11on, k11off, kd22, kd23_2, kd23p_1, w4, kon_BTK, koff_BTK, w16, w17, umax, dmax, w11, w12, w13, w15, w14 (see Supplementary Table S1 for more details of these parameters).

**
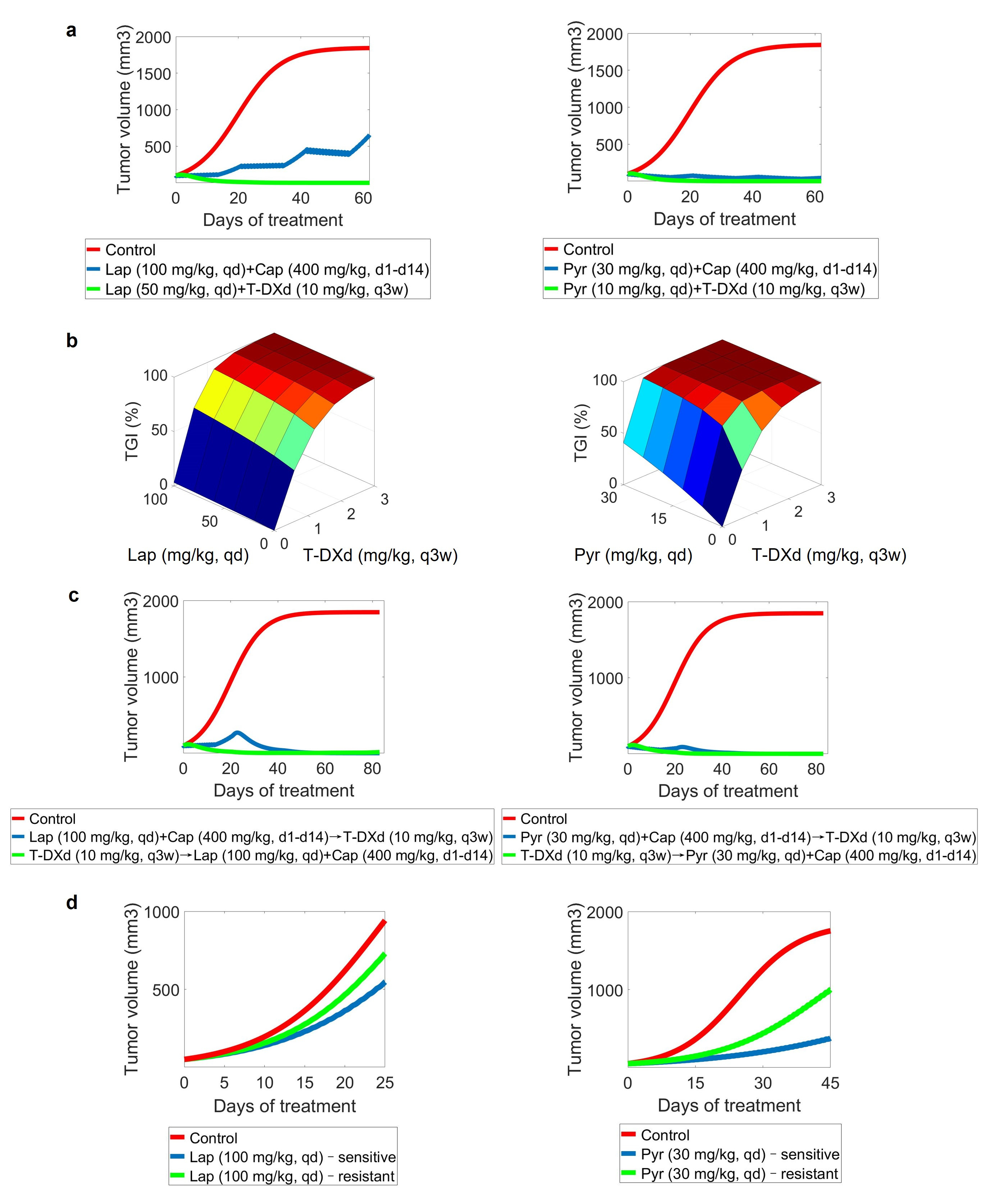
**

**Supplementary Fig. S7 Additional model evaluations of tumor response kinetics in vivo under different treatment strategies. See also Fig. 7. a** Simulated antitumor effect of the new combination regimen of lapatinib or pyrotinib plus T-DXd versus classic lapatinib or pyrotinib plus capecitabine. **b** Simulated tumor growth inhibition (TGI) to combinations of lapatinib or pyrotinib with T-DXd over a range of doses (lapatinib 20–100 mg/kg qd, pyrotinib 6–30 mg/kg qd, and T-DXd 0.6–3 mg/kg q3w, respectively). Tumor volumes were analyzed after three treatment cycles (e.g., on day 62). **c** Simulated antitumor effects of sequential therapies of lapatinib or pyrotinib plus capecitabine followed by T-DXd or T-DXd followed by lapatinib or pyrotinib plus capecitabine. **d** Simulated tumor kinetics in acquired TKI resistance, with the assumption that cumulative drug exposure may result in a decrease in therapeutic potency (realized by setting *km*, Hill function of TKI to its target, as proportional to time). See the legends for the detailed dosage and frequency of administrations, where a treatment cycle is 21 days and d1–d14 means capecitabine is administered on days 1–14 of each cycle.

**
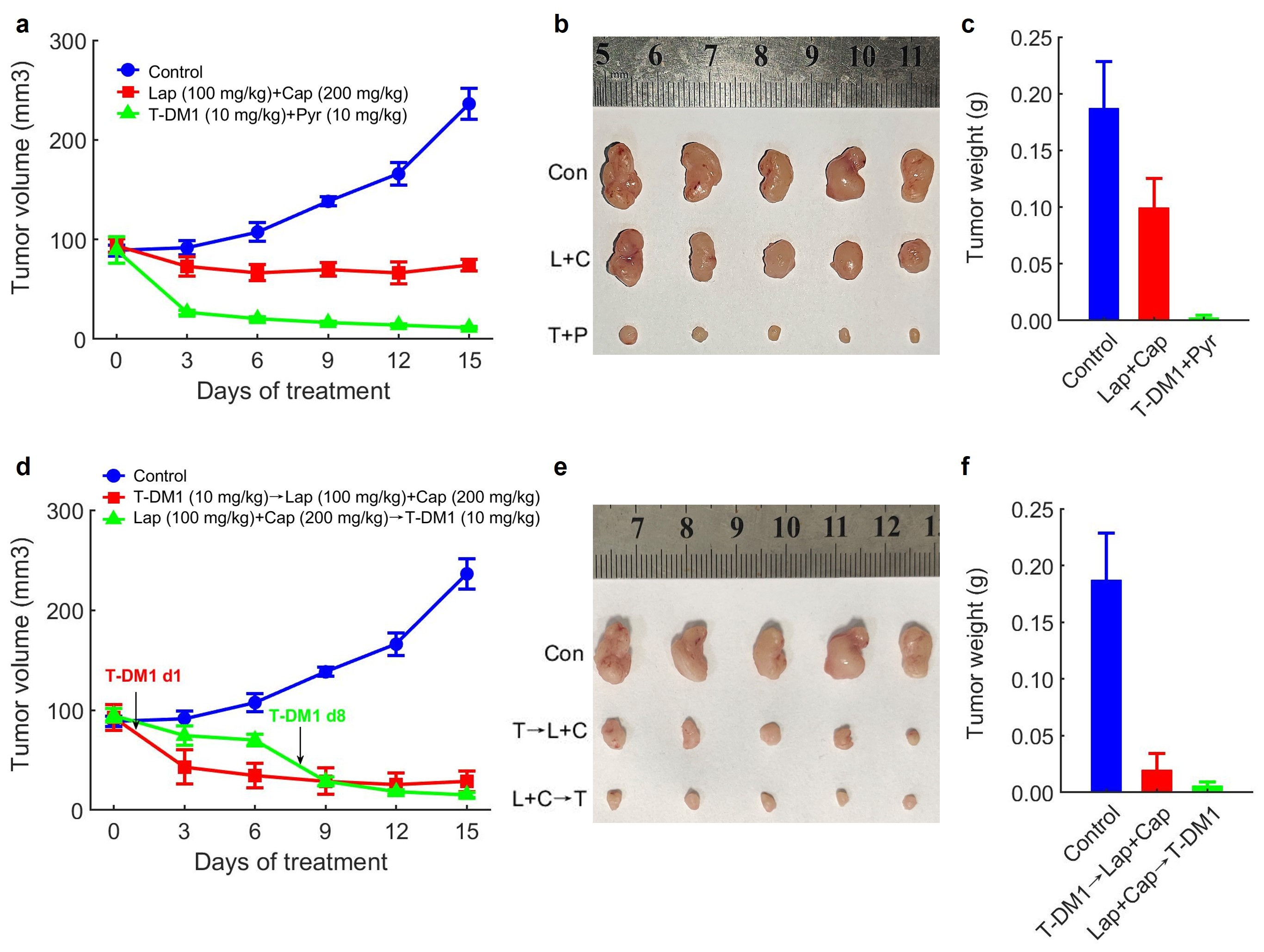
**

**Supplementary Fig. S8 In vivo experimental validation of model-predicted tumor growth kinetics in response to combination or sequential therapies. See also Fig. 7.** Quantified tumor growth curves, photos of the excised tumors and tumor weight of mice that received combination therapies **(a**–**c)** or sequential therapies **(d**–**f)** (*n*=5 for each arm). Tumor photos and weights were obtained at the end of treatment, and the same time-course tumor growth data from control group were used for comparison in a–f. Con, control; L, lapatinib; C, capecitabine; T, T-DM1; P, pyrotinib.

**
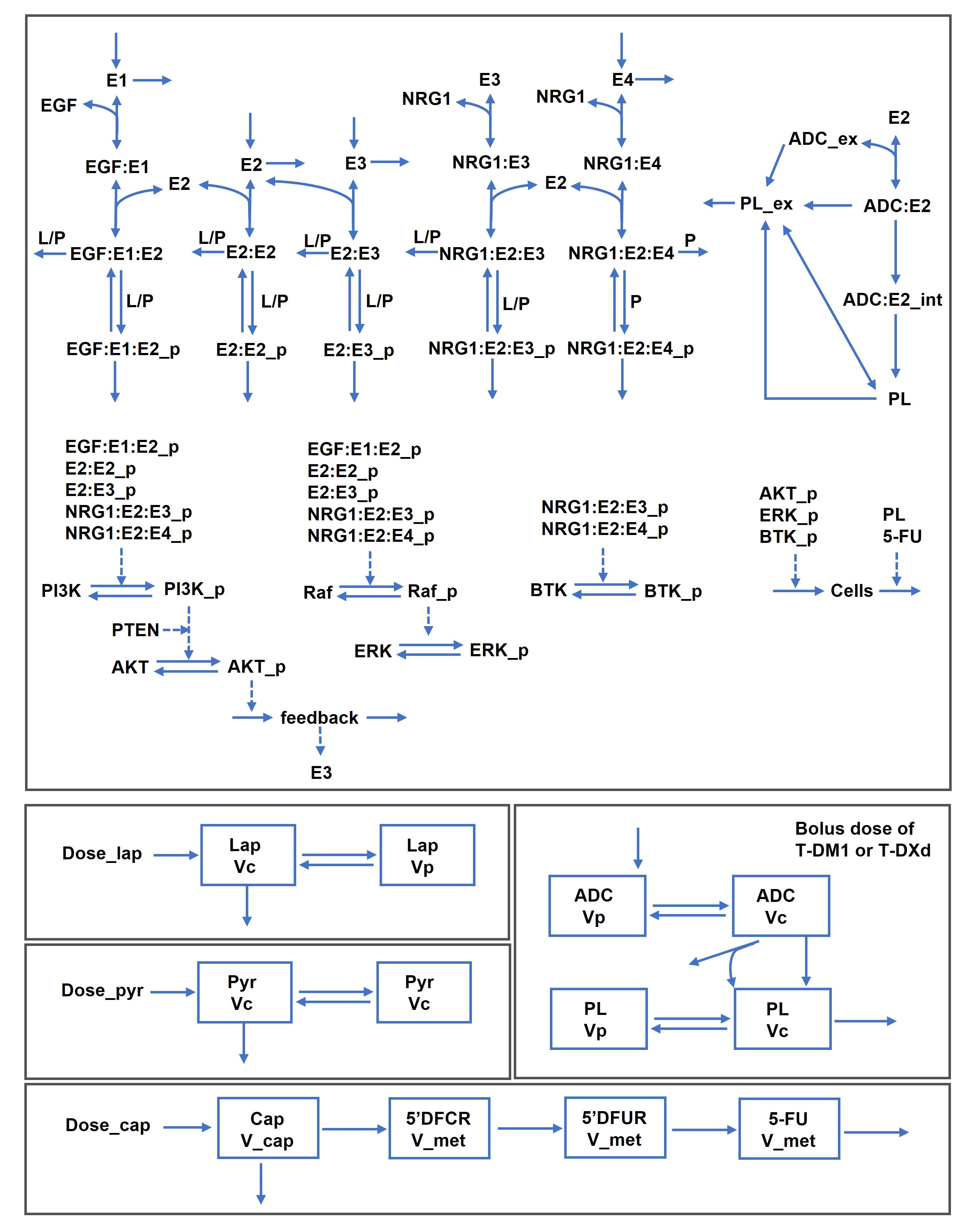
**

**Supplementary Fig. S9 Detailed model diagram. See also Fig. 1.** A detailed model diagram with specific model species and reaction fluxes, along with pharmacokinetic details of five drugs, including lapatinib, pyrotinib, capecitabine, T-DM1 and T-DXd. For a complete description of all species, parameters and reactions, please refer to Supplementary Table S1. L, lapatinib; P, pyrotinib.

**Supplementary Table S2.** Comparison of model-predicted TGI and Bliss-predicted TGI of four drug combination regimens.

| **TGI**  **Lap** | **0** | **2** | **4** | **6** | **8** | **10** |
| --- | --- | --- | --- | --- | --- | --- |
| **0** | 0 | 2.32 | 10.04 | 25.58 | 50.84 | 77.66 |
| **20** | 0.86 | 3.78/3.16 | 13.71/10.81 | 33.89/26.22 | 63.86/51.27 | 87.27/77.86 |
| **40** | 1.57 | 4.96/3.85 | 16.62/11.45 | 40.31/26.74 | 71.78/51.61 | 91.41/78.01 |
| **60** | 2.16 | 5.99/4.43 | 19.16/11.98 | 45.55/27.19 | 77.06/51.90 | 93.65/78.15 |
| **80** | 2.75 | 6.91/5.01 | 21.42/12.51 | 49.95/27.63 | 80.78/52.19 | 95.01/78.28 |
| **100** | 3.24 | 7.77/5.48 | 23.53/12.95 | 53.71/27.99 | 83.53/52.43 | 95.93/78.39 |

**T-DM1**

| **TGI**  **Pyr** | **0**  **T-DM1** | **2** | **4** | **6** | **8** | **10** |
| --- | --- | --- | --- | --- | --- | --- |
| **0** | 0 | 2.32 | 10.04 | 25.58 | 50.84 | 77.66 |
| **6** | 11.23 | 20.02/13.29 | 48.16/20.14 | 81.57/33.93 | 96.01/56.36 | 99.21/80.17 |
| **12** | 20.08 | 32.00/21.93 | 64.20/28.10 | 90.29/40.52 | 98.17/60.71 | 99.65/82.15 |
| **18** | 27.90 | 41.55/29.57 | 73.29/35.14 | 93.66/46.34 | 98.87/64.56 | 99.79/83.90 |
| **24** | 34.75 | 49.16/36.27 | 79.02/41.30 | 95.40/51.44 | 99.20/67.93 | 99.85/85.43 |
| **30** | 40.74 | 55.30/42.12 | 82.91/46.69 | 96.44/55.90 | 99.39/70.87 | 99.89/86.76 |

**T-DXd**

| **TGI**  **Lap** | **0** | **0.6** | **1.2** | **1.8** | **2.4** | **3** |
| --- | --- | --- | --- | --- | --- | --- |
| **0** | 0 | 47.05 | 76.71 | 91.31 | 97.02 | 98.92 |
| **20** | 0.86 | 52.32/47.51 | 82.49/76.91 | 94.64/91.38 | 98.41/97.04 | 99.47/98.93 |
| **40** | 1.57 | 56.07/47.88 | 85.89/77.08 | 96.18/91.44 | 98.95/97.06 | 99.66/98.94 |
| **60** | 2.16 | 59.04/48.20 | 88.19/77.22 | 97.06/91.49 | 99.23/97.08 | 99.76/98.94 |
| **80** | 2.75 | 61.53/48.51 | 89.85/77.35 | 97.63/91.55 | 99.39/97.10 | 99.81/98.95 |
| **100** | 3.24 | 63.67/48.77 | 91.11/77.47 | 98.02/91.59 | 99.51/97.11 | 99.85/98.96 |

| **TGI**  **T-DXd**  **Pyr** | **0** | **0.6** | **1.2** | **1.8** | **2.4** | **3** |
| --- | --- | --- | --- | --- | --- | --- |
| **0** | 0 | 47.05 | 76.71 | 91.31 | 97.02 | 98.92 |
| **6** | 11.23 | 81.60/53.00 | 97.45/79.33 | 99.56/92.28 | 99.90/97.35 | 99.97/99.04 |
| **12** | 20.08 | 89.02/57.68 | 98.76/81.39 | 99.80/93.05 | 99.96/97.62 | 99.99/99.14 |
| **18** | 27.90 | 92.37/61.83 | 99.22/83.21 | 99.88/93.73 | 99.97/97.85 | 99.99/99.22 |
| **24** | 34.75 | 94.26/65.45 | 99.44/84.81 | 99.91/94.33 | 99.98/98.05 | 99.99/99.30 |
| **30** | 40.74 | 95.44/68.63 | 99.57/86.20 | 99.93/94.85 | 99.99/98.23 | 100/99.36 |

TGI: tumor growth inhibition (%)

Lap: lapatinib (mg/kg); Pyr: pyrotinib (mg/kg); T-DM1: (mg/kg); T-DXd: (mg/kg)

The cells under each dose pair show two numbers separated by a slash. The number to the left of the slash is model-predicted TGI and the number to the right of the slash is Bliss-predicted TGI.

**References**

1. Selitrennik M, Lev S. PYK2 integrates growth factor and cytokine receptors signaling and potentiates breast cancer invasion via a positive feedback loop. Oncotarget. 2015; 6: 22214-22226.

2. Novotny CJ, Pollari S, Park JH, Lemmon MA, Shen WJ, Shokat KM. Overcoming resistance to HER2 inhibitors through state-specific kinase binding. Nat Chem Biol. 2016; 12: 923-930.

3. Li GY, Wang XQ, Hibshoosh H, Jin C, Halmos B. Modulation of ErbB2 blockade in ErbB2-positive cancers: the role of ErbB2 mutations and PHLDA1. PLoS One. 2014; 9: e106349.

4. Amin DN, Sergina N, Ahuja D, McMahon M, Blair JA, Wang DH, et al. Resiliency and vulnerability in the HER2-HER3 tumorigenic driver. Sci Transl Med. 2010; 2: 16ra17.

5. Chen CH, Hsia TC, Yeh MH, Chen TW, Chen YJ, Chen JT, et al. MEK inhibitors induce Akt activation and drug resistance by suppressing negative feedback ERK‐mediated HER2 phosphorylation at Thr701. Mol Oncol. 2017; 11: 1273-1287.

6. Zhang K, Hong RX, Kaping L, Xu F, Xia W, Qin G, et al. CDK4/6 inhibitor palbociclib enhances the effect of pyrotinib in HER2-positive breast cancer. Cancer Lett. 2019; 447: 130-140.

7. Yi JN, Chen S, Yi PY, Luo JL, Fang M, Du Y, et al. Pyrotinib sensitizes 5-fluorouracil-resistant HER2+ breast cancer cells to 5-fluorouracil. Oncol Res. 2020; 28: 519-531.

8. Berry L, Phillips GL, Sliwkowski MX. Combinations of an anti-HER2 antibody-drug conjugate and lapatinib, and methods of use. patent EP3269366. 2018 Jan 17.

9. Bu JW, Zhang YX, Niu N, Bi KW, Sun LS, Qiao XB, et al. Dalpiciclib and pyrotinib exert synergistic antitumor effects in triple positive breast cancer. bioRxiv. 2021 04 Oct. Doi: 10.1101/2021.10.04.463019.

10. Yin T, Wang RR, Yang SZ. Anti-breast cancer activity of Co(II) complex by inhibiting cell viability and stimulating cell apoptosis. J Clust Sci. 2021; 33: 2763-2770.

11. Nonagase Y, Yonesaka K, Kawakami H, Watanabe S, Haratani K, Takahama T, et al. Heregulin-expressing HER2-positive breast and gastric cancer exhibited heterogeneous susceptibility to the anti-HER2 agents lapatinib, trastuzumab and T-DM1. Oncotarget. 2016; 7: 84860-84871.

12. Su BB, Huang TT, Jin Y, Yin H, Qiu H, Yuan XL. Apatinib exhibits synergistic effect with pyrotinib and reverses acquired pyrotinib resistance in HER2-positive gastric cancer via stem cell factor/c-kit signaling and its downstream pathways. Gastric Cancer. 2021; 24: 352-367.

13. Nakamura A, Nakajima G, Okuyama R, Kuramochi H, Kondoh Y, Kanemura T, et al. Enhancement of 5-fluorouracil-induced cytotoxicity by leucovorin in 5-fluorouracil-resistant gastric cancer cells with upregulated expression of thymidylate synthase. Gastric Cancer. 2014; 17: 188-195.

14. Zhang JH, Fan JJ, Zeng X, Nie MM, Chen W, Wang YC, et al. Targeting the autophagy promoted antitumor effect of T-DM1 on HER2-positive gastric cancer. Cell Death Dis. 2021; 12: 288.

15. van Agthoven T, Dorssers LC, Lehmann U, Kreipe H, Looijenga LH, Christgen M. Breast cancer anti-estrogen resistance 4 (BCAR4) drives proliferation of IPH-926 lobular carcinoma cells. PLoS One. 2015; 10: e0136845.

16. Tsao DA, Chang HJ, Lin CY, Hsiung SK, Huang SE, Ho SY, et al. Gene expression profiles for predicting the efficacy of the anticancer drug 5-fluorouracil in breast cancer. DNA Cell Biol. 2010; 29: 285-293.

17. van der Lee MM, Groothuis PG, Ubink R, van der Vleuten MA, van Achterberg TA, Loosveld EM, et al. The preclinical profile of the duocarmycin-based HER2-targeting ADC SYD985 predicts for clinical benefit in low HER2-expressing breast cancers. Mol Cancer Ther. 2015; 14: 692-703.

18. Chakrabarty A, Sanchez V, Kuba MG, Rinehart C, Arteaga CL. Feedback upregulation of HER3 (ErbB3) expression and activity attenuates antitumor effect of PI3K inhibitors. Proc Natl Acad Sci U S A. 2012; 109: 2718-2723.
