# Supplementary material for: Quantitative systems pharmacology modeling of HER2-positive metastatic breast cancer for translational efficacy evaluation and combination assessment across therapeutic modalities
Source: Acta Pharmacol Sin. 2024 Feb 15;45(6):1287–304. doi: 10.1038/s41401-024-01232-9 (PMC11130324; doi:10.1038/s41401-024-01232-9)
Supplement: Supplementary file 3 — Supplementary Table S1 legend [file 41401_2024_1232_MOESM3_ESM.docx]

**Supplementary Table S1 (Excel).** A summary of all model species, parameters, reactions and their descriptions, along with a sheet summarizing data used for calibration and validation.
